# Powdered Herbal Extracts by Freeze and Spray Drying: Formulation Design, Processing, and Physicochemical and Antiglycation Properties

**DOI:** 10.3390/foods15193371

**Published:** 2026-09-22

**Authors:** Anna Plaskova, Jiri Mlcek, Anna Michalska-Ciechanowska

**Affiliations:** 1 Department of Food Analysis and Chemistry, Faculty of Technology, Tomas Bata University in Zlin, Vavreckova 5669, 760 01 Zlin, Czech Republic; 2 Department of Fruit, Vegetable and Plant Nutraceutical Technology, Faculty of Biotechnology and Food Science, Wrocław University of Environmental and Life Sciences, Chełmońskiego 37 Str., 51-630 Wrocław, Poland; anna.michalska@upwr.edu.pl

**Keywords:** *Alchemilla vulgaris*, *Mentha × piperita*, *Urtica dioica*, aqueous extraction, herbal powders, carrier agents, inulin, phenolic compounds, antioxidant capacity, antiglycation activity

## Abstract

Drying and carrier effects may differ among botanical materials, but direct comparisons remain limited. This study compared spray drying (SD) and freeze-drying (FD) across four extract formulations from *Alchemilla vulgaris*, *Mentha × piperita*, *Urtica dioica*, and two 1:1 *A. vulgaris*-based binary mixtures. Each extract was dried without a carrier or with 5% (*w/w*) maltodextrin, inulin, or a 95:5 inulin-pea-protein isolate blend. Powders were evaluated for process efficiency, moisture content, water activity, total phenolics content (TPC), antioxidant capacity, and apparent inhibition of fluorescent advanced glycation end products (AGE) formation in four in vitro glycation models. SD powders had lower moisture content than FD powders (1.13–4.18% vs. 4.32–10.49%), whereas FD provided higher process efficiency (74.8–98.1% vs. 47.2–82.0%). Carrier-free powders generally had higher water activity, TPC, and antioxidant capacity. TPC ranged from 3.31 to 36.99 g gallic acid equivalents/100 g dry matter, compared with 0.63–3.66 in carrier-containing powders. Mean apparent AGE inhibition of carrier-free powders exceeded that of carrier-containing formulations by 16.9–37.9 percentage points. Botanical rankings were model-dependent: *M. piperita* was strongest in the BSA–glucose model and *A. vulgaris* in the BSA–methylglyoxal model. Carrier selection therefore requires balancing process efficiency and water activity against TPC and apparent AGE inhibition.

## 1. Introduction

Herbs are complex plant matrices containing a chemically diverse range of secondary metabolites. Among these, phenolic compounds form one of the most prominent classes, encompassing phenolic acids, flavonoids, tannins, stilbenes, and lignans [1,2]. This group of compounds can influence the color, bitterness, and astringency of plant-derived foods and beverages. Astringency results primarily from non-covalent interactions between higher-molecular-weight polyphenols and proline-rich salivary proteins [3,4]. Aqueous herbal extracts containing phenolic compounds have also been investigated as potential functional food ingredients [5,6]. Their phytochemical composition can nonetheless vary depending on the extraction solvent, extraction temperature, duration, and drying process used to produce powdered extracts [7].

*Alchemilla vulgaris* L. (lady’s mantle; *Rosaceae*), *Mentha × piperita* L. (peppermint; *Lamiaceae*), and *Urtica dioica* L. (stinging nettle; *Urticaceae*) represent taxonomically distinct herbs with a long history of traditional use [8,9,10,11]. Their aqueous extracts have been reported to contain phenolic compounds with relatively strong antioxidant activity [10,11,12,13,14]. Antiglycation activity has also been reported for *M. piperita* and *U. dioica*. A dry extract prepared from a peppermint water infusion inhibited methylglyoxal-induced glycation of bovine serum albumin in vitro, with eriocitrin estimated to make the largest contribution to the observed activity [9]. Aqueous *U. dioica* leaf extracts likewise inhibited methylglyoxal-induced glycation of bovine serum albumin in vitro [15]. In the related species *Alchemilla mollis*, an aqueous leaf extract showed higher mean inhibition of advanced glycation end product formation in a bovine serum albumin–glucose model (BSA–Glu) than the corresponding methanolic and dichloromethane extracts [16]. Pedunculagin and agrimoniin have been identified as major phenolic constituents of both *A. vulgaris* and *A. mollis* following aqueous acetone extraction, although the two species differed in other constituents [17]. This compositional overlap provides a limited basis for comparison, but the antiglycation activity of *A. mollis* cannot be directly extrapolated to *A. vulgaris*.

The selection of an extraction method and operating conditions should account for the characteristics of both the plant matrix and the target compounds [18]. More specifically, the phytochemical composition and yield of an aqueous herbal extract depend on the herb/water ratio, extraction temperature, and time. Insufficient heating may limit the recovery of bioactive compounds, whereas excessive temperatures or prolonged extraction can promote the degradation of thermolabile phenolic constituents [1,2]. Even under optimized extraction conditions, an aqueous herbal extract is mostly water. This may limit its practical use: the extract requires considerable storage volume relative to its phenolic content, has limited stability over time, and is difficult to dose precisely in food formulations [19]. Drying removes most of this water, yielding a concentrated, food-grade powder that is easier to store, dose, and incorporate into formulations; this conversion is commonly achieved by combining the extract with a carrier agent before drying [20,21]. The aqueous extraction conditions were therefore adjusted before the extracts were processed into powders in the present study.

Spray drying (SD) and freeze-drying (FD) are widely used techniques to convert liquid plant extracts into powders for food and pharmaceutical applications [22]. The two processes remove water by fundamentally different mechanisms [23,24]. During SD, the liquid extract is atomized into hot air and rapidly dried, typically forming small, largely spherical particles [20,25]. In FD, water is removed from a frozen matrix by sublimation under vacuum. The resulting powders generally have a more porous and irregular structure than their spray-dried counterparts [23,24]. The relatively low temperatures used during FD may help preserve thermolabile bioactive compounds [22,26]. Spray drying, by contrast, is considerably faster and generally better suited to industrial-scale production [20,25]. Neither SD nor FD has been consistently superior across the powder properties evaluated [24,27,28], and their relative effects have varied with feed-matrix composition and carrier formulation [22,29].

Carriers are often added before drying to facilitate powder production and improve process yield [22]. They are particularly useful when a drying feed contains substantial amounts of low-molecular-weight sugars and organic acids [29,30,31]. During storage, moisture uptake can lower *T_g_* and thereby reduce the physical stability of the powder [27]. Beyond their effect on drying behavior, carriers may alter powder properties, bioactive-compound retention, and measured antioxidant capacity [22,30].

Maltodextrin, a starch-derived carbohydrate carrier with a neutral taste, is widely used because it dissolves readily in water and forms low-viscosity solutions even at high solid concentrations [28,32]. It also serves as a conventional comparator in studies evaluating alternative carrier systems [28,30,33,34], and this carrier was therefore selected as the technological reference in the present study. Inulin, a fructan-based carrier, was selected because it can vitrify during drying, forming a noncrystalline glassy matrix. This matrix remains rigid below its glass transition temperature and may improve the physical stability of dried products [35,36]. It has also been evaluated as a spray drying carrier for aqueous herbal extracts prepared from *Cyclopia subternata* and *Hibiscus sabdariffa* L. [37,38]. In addition to its technological role, inulin is a non-digestible dietary fiber with prebiotic properties and may therefore provide nutritional functionality [35,38].

Besides polysaccharides, proteins can also serve as carriers, since their amphiphilic and film-forming properties may complement the matrix-forming function of carbohydrates [39,40]. Pea protein isolate (PPI), derived from the seeds of *Pisum sativum* L. and composed mainly of the globulins legumin and vicilin, was selected as a plant-based alternative to soy with relatively low allergenic potential [39,41]. However, its functionality (solubility, gel formation, emulsification) depends strongly on pH and ionic strength [39]. Limited solubility and high viscosity at high concentrations can restrict its use as a sole wall material [32], while characteristic beany off-flavors may reduce its sensory acceptability [42]. Combining PPI with polysaccharides has been reported to address these limitations [32] and to reduce off-flavors by altering protein secondary structure [42]. PPI has previously been combined with carbohydrate carriers in maltodextrin–PPI carriers for spray- and freeze-dried *Echinacea purpurea* root extract [28] in spray-dried fat-filled formulations with inulin [43]. PPI-stabilized oil emulsions containing starch hydrolysates have also been spray-dried using 0.25% (*w/w*) PPI in the pre-drying emulsion [44]. The final inulin–PPI feed formulation in the present study likewise contained 0.25% (*w/w*) PPI.

Previous powder-production studies involving peppermint and nettle have used different feed matrices, carrier systems, and drying conditions [45,46,47,48,49,50,51,52]. None of the publications examined addressed powders prepared from binary mixtures of *A. vulgaris* with *M. piperita* or *U. dioica*. These research papers also did not compare spray drying and freeze-drying of the same aqueous extracts across carrier-free, maltodextrin-, inulin-, and inulin-PPI-based formulations while jointly assessing physicochemical powder properties, phenolic content, antioxidant capacity, and in vitro antiglycation activity.

Alongside this technological gap, the retention of biological activity under different drying and carrier conditions also remains insufficiently characterized. Non-enzymatic glycation begins when reducing sugars, such as glucose and fructose, or reactive carbonyl species react with nucleophilic amino or guanidino groups in proteins [9,53]. In reactions initiated by glucose, unstable Schiff bases are first formed and subsequently rearranged into more stable Amadori products [54]. Further oxidation, dehydration, and condensation reactions can generate a heterogeneous group of compounds known as advanced glycation end products (AGEs) [54]. Among the reactive carbonyl species involved, methylglyoxal (MGO) is a highly reactive α-dicarbonyl compound and an important precursor of AGE formation [9,54]. AGE accumulation has been associated with aging, oxidative stress, and several metabolic disorders, although the causal nature of some of these relationships remains unresolved [53]. These associations have stimulated interest in food-derived compounds that may interfere with AGE formation [54].

Plant phenolics may interfere with AGE formation by scavenging free radicals, chelating metals, and trapping reactive dicarbonyl compounds such as MGO [54,55]. Inhibition of AGE formation has been reported to differ among botanical extracts and glycation models [56,57]. Its association with antioxidant capacity also varies among the preparations examined, supporting the evaluation of antioxidant and antiglycation activity as separate outcomes [58,59].

Accordingly, the primary aim of this study was to compare the effects of drying technique and carrier formulation on processing performance, physicochemical powder properties, phenolic content, antioxidant capacity, and in vitro antiglycation activity of powders prepared from aqueous extracts of *A. vulgaris*, *M. piperita*, *U. dioica*, and two *A. vulgaris*-based binary mixtures. Each extract was spray-dried and freeze-dried using four formulations: carrier-free, maltodextrin, inulin, and an inulin–PPI blend. Antioxidant capacity and antiglycation activity were assessed as separate endpoints, with the latter evaluated using four glycation models to examine model-dependent responses. As a secondary objective, this study examined whether *A. vulgaris* exhibited measurable antiglycation activity under the same standardized assay conditions used for *M. piperita* and *U. dioica*. We hypothesized that drying technique and carrier formulation would affect powder properties and measured bioactivity and that *A. vulgaris* would show apparent inhibition of AGE formation in the tested in vitro models. To our knowledge, this is the first study to directly assess the in vitro antiglycation activity of *A. vulgaris* under standardized conditions.

## 2. Materials and Methods

### 2.1. Plant Materials and Carrier Agents

Commercial dried, cut herbal materials were purchased from Grešík (Děčín, Czech Republic) in April 2023, each supplied in a 1 kg package. The materials comprised aerial parts of *Alchemilla vulgaris* L. (leaves and stems; batch no. 070122/070323; country of origin: Czech Republic), *Mentha × piperita* L. leaves (batch no. 020622/020323; country of origin: Poland), and *Urtica dioica* L. leaves (batch no. 280222/081122; country of origin: Slovakia). According to information provided by the supplier, all materials were harvested in 2021. Two binary herbal mixtures, *A. vulgaris* + *M. piperita* (AV + MP) and *A. vulgaris* + *U. dioica* (AV + UD), were prepared by combining 25 g of each constituent herb, yielding 50 g mixtures with a 1:1 (*w/w*) composition. The mixtures were placed in sealed glass jars and shaken for 4 min to distribute the constituent materials throughout the mixture. The moisture content of the single-herb samples ranged from 7.07 ± 0.08% for *U. dioica* to 9.18 ± 0.08% for *M. piperita*, while the *A. vulgaris* specimen exhibited a moisture content of 7.16 ± 0.01%. The moisture contents of the AV + UD and AV + MP mixtures were 6.99 ± 0.13% and 8.10 ± 0.10%, respectively.

Maltodextrin (Przedsiębiorstwo Przemysłu Spożywczego PEPEES S.A., Łomża, Poland) and inulin powder (GR type, Beneo-Orafti^®^ S.A., Oreye, Belgium) were used as carrier agents, together with a combination of inulin and pea protein isolate (5%, *w/w*, of the carrier blend; Myvegan, Manchester, United Kingdom). An aliquot of each herbal extract without an added carrier served as the carrier-free control (C).

### 2.2. Chemicals and Reagents

Methanol (p.a.; P.P.H. “STANLAB” Sp. z o.o., Lublin, Poland) was used for the preparation of methanolic powder extracts.

Reagents for the total phenolics content and antioxidant assays comprised Folin–Ciocalteu reagent (Chempur, Piekary Śląskie, Poland), anhydrous sodium carbonate (P.P.H. “STANLAB” Sp. z o.o., Lublin, Poland), gallic acid (≥98%; Carl Roth GmbH + Co. KG, Karlsruhe, Germany), Fast Blue BB salt hemi(zinc chloride) (≥80%; Sigma-Aldrich Chemie GmbH, Steinheim, Germany), sodium hydroxide granules (p.a.; P.P.H. “STANLAB” Sp. z o.o., Lublin, Poland), 2,2′-azino-bis(3-ethylbenzothiazoline-6-sulfonic acid) diammonium salt (ABTS; Sigma-Aldrich Chemie GmbH, Steinheim, Germany), potassium peroxodisulfate (p.a., ≥99.0%; Sigma-Aldrich Chemie GmbH, Steinheim, Germany), Trolox (97%; Thermo Fisher Scientific, Geel, Belgium), 2,4,6-tris(2-pyridyl)-s-triazine (TPTZ; ≥98%; Sigma-Aldrich Chemie GmbH, Steinheim, Germany), sodium acetate anhydrous (p.a.; Chempur, Piekary Śląskie, Poland), hydrochloric acid (35–38%; P.P.H. “STANLAB” Sp. z o.o., Lublin, Poland), and iron(III) chloride hexahydrate (p.a.; Chempur, Piekary Śląskie, Poland).

Reagents for the antiglycation assays comprised bovine serum albumin (BSA; ≥99%; Pol-Aura, Dywity, Poland), D-glucose anhydrous (p.a.; Chempur, Piekary Śląskie, Poland), D-fructose (p.a.; Chempur, Piekary Śląskie, Poland), methylglyoxal solution (MGO; ~40% in H_2_O; Sigma-Aldrich Chemie GmbH, Steinheim, Germany), L-arginine (reagent grade, ≥98%; Sigma-Aldrich Chemie GmbH, Steinheim, Germany), aminoguanidine hydrochloride (≥98%; Sigma-Aldrich Chemie GmbH, Steinheim, Germany), sodium azide (p.a.; Chempur, Piekary Śląskie, Poland), di-sodium hydrogen phosphate anhydrous (p.a.; Chempur, Piekary Śląskie, Poland), and sodium dihydrogen phosphate monohydrate (p.a.; Chempur, Piekary Śląskie, Poland).

### 2.3. Preliminary Selection of Aqueous Extraction Conditions

Before the preparation of the extracts used for powder production, preliminary trials were conducted to select an herb/water ratio and extraction time. Dried herbal material in its as-received, unground form (1, 2, or 3 g) was combined with approximately 100 g of water preheated to 90 °C. The covered beakers were placed in a water bath (GFL type 1023; Gesellschaft für Labortechnik mbH, Burgwedel, Germany) maintained at 90 °C for up to 120 min. Aliquots (1.5 mL) were collected immediately after the addition of water (t = 0) and after 15, 30, 60, and 120 min. The samples were frozen right away and stored until analysis.

Two independent extractions (*n* = 2) were performed for each condition, and each extract was analyzed in duplicate. After thawing, the samples were centrifuged at 14,000 rpm for 7 min using an MPW M-UNIVERSAL centrifuge (MPW MED. INSTRUMENTS, Warsaw, Poland), and the resulting supernatants were used to determine total phenolics content.

Based on the resulting extraction profiles, an herb/water ratio of 2 g per 100 g of water and an extraction time of 60 min at 90 °C were selected as common preparation conditions for all single-herb extracts and binary mixtures. These conditions were not individually optimized for each herb or mixture but represented a suitable compromise across all of them.

### 2.4. Preparation of Herbal Extracts and Binary Herbal Mixtures

Using the herb/water ratio, temperature, and extraction time selected in Section 2.3, each aqueous extract was prepared by combining 8 g of as-received, unground herbal material with approximately 400 g of preheated distilled water. After extraction, the liquid was rapidly cooled and strained through a cloth filter, and the retained plant material was manually pressed to recover the remaining extract.

For the binary extracts, an 8 g portion of each premixed binary herbal material described in Section 2.1 was extracted under the same conditions as the single-herb materials.

### 2.5. Carrier Addition

The extracts (prepared as described in Section 2.4) were cooled to room temperature to limit evaporation and ensure accurate mass measurements. Each herbal extract was divided into four portions. Inulin (IN), maltodextrin (MD), or a 95:5 (*w/w*) inulin-pea-protein-isolate blend (IN + PPI) was incorporated to a final concentration of 5% (*w/w*) based on the total weight of the final extract–carrier mixture (e.g., 5 g of carrier added to 95 g of liquid extract). For the carrier-free control (C), an equivalent mass of distilled water was used instead of carrier. The carriers were dispersed by gentle mixing with a glass stirring rod until the mixtures appeared homogeneous and no visible sediment remained. Dissolution required approximately 10 to 20 min, with certain formulations requiring continuous mixing until no visible sediment remained and the mixture appeared homogenous.

### 2.6. Freeze-Drying

The extract–carrier mixtures were transferred into pre-weighed crystallizing dishes and frozen at −18 °C for at least 1 day prior to drying. FD was then carried out under reduced pressure for 24 h using a FreeZone freeze dryer (Labconco Corp., Kansas City, MO, USA) at a chamber pressure of 65 Pa, with the condenser temperature maintained at −60 °C and the heating shelves at approximately 24–25 °C. The drying was performed in three technological replicates (*n* = 3).

### 2.7. Spray Drying

Spray drying (SD) was performed using a Mini Spray Dryer B-290 (Büchi Labortechnik AG, Flawil, Switzerland) operated at an inlet temperature of 160 °C and an outlet temperature of 94–98 °C. The aspirator was set to 100%, the feed rate to 4 mL/min (pump at 15%), and the nozzle-cleaning frequency to 5. The drying was performed in two technological replicates (*n* = 2).

### 2.8. Powder Packaging and Storage

After the equipment had cooled, freeze-dried powders were ground to a homogeneous powder with a mortar and pestle, and both freeze-dried and spray-dried powders were swept with a brush into pre-weighed bags; the filled bags were reweighed on an analytical balance (AS 110/C/2; RADWAG Wagi Elektroniczne, Radom, Poland) to determine the process efficiency (Section 2.10). Powders were vacuum-packed into moisture-resistant polyamide/polyethylene bags using a vacuum packing machine (PP 4.2E; ZTP TEPRO S.A., Koszalin, Poland) and stored at −20 °C until analysis.

### 2.9. Determination of Soluble Solids and Dry Matter

Soluble solids content (°Brix) of the liquid extracts and carrier-assisted mixtures was measured with a pocket digital Brix refractometer (PAL-1; Atago, Tokyo, Japan), as described in Section 2.4 and Section 2.5.

To determine the dry matter of the raw plant material, the herbs were ground into a homogeneous powder using a coffee grinder (type KM13; Robert Bosch Hausgeräte GmbH, Munich, Germany), and 0.2–0.3 g of sample was weighed into pre-dried, pre-weighed glass weighing bottles (dried at 110 °C for 2 h, 500 W). Samples were then dried at 80 °C for 3 h at a pressure of 1 kPa in a vacuum dryer (Spt-200; ZEAMiL HORYZONT, Krakow, Poland), cooled in a desiccator, and reweighed to four decimal places on an analytical balance. Measurements were performed in duplicate (*n* = 2).

The dry matter of the liquid extract–carrier mixtures and the resulting powders was determined using an analogous procedure, except that 0.2–0.4 g of powder, or an 800 μL aliquot of the extract–carrier mixture pipetted onto pre-dried, acid-washed diatomaceous earth in a weighing bottle containing a glass stirring rod, was dried at 70 °C for 20 h in a vacuum dryer (Vacucell 111 Eco line; MMM Medcenter Einrichtungen GmbH, Planegg, Germany). Weighing bottles were cooled in a desiccator and reweighed as above. The moisture content of the final powders was calculated as the complement of the dry matter determined by this procedure.

### 2.10. Process Efficiency

The process efficiency of both spray- and freeze-drying was determined gravimetrically from the mass of total solids present in the sprayed or dosed extract–carrier mixture and the mass of dry powder recovered at the end of the process, and was expressed as a percentage:

(1) $$\mathrm{Process efficiency}\left(\%\right)=\frac{\mathrm{mass of powder}(g)\times 100}{\mathrm{solids content of the extract}-\mathrm{carrier mixture}(g)},$$

where the mass of powder is the mass recovered after drying (Section 2.8), and the solids content of the mixture is derived from the dry matter determination described in Section 2.9.

### 2.11. Water Activity

The water activity (*a_w_*) of the powders was measured at 25 °C using a Dew Point Water Activity Meter 4TE (METER Group, Inc., Pullman, WA, USA), with each powder measured in duplicate (*n* = 2).

### 2.12. Color Parameters and Browning Index

The color of the powders was measured in triplicate (*n* = 3) using a Spectrophotometer CM-700d (Konica Minolta, Inc., Osaka, Japan) according to the CIE L*a*b* color system, in which L* quantifies lightness (0 = black, 100 = white), a* spans green (−a*) to red (+a*), and b* spans blue (−b*) to yellow (+b*).

The browning index (BI) was calculated from the CIE L*a*b* coordinates using the equation of Palou et al. (1999) [60]:

(2) BI=x−0.31×1000.172,where x=a*+1.75×L*5.645×L*+a*−3.012×b*.

### 2.13. Determination of Total Phenolics Content

Prior to chemical analysis, powder extracts were prepared by dissolving 20 or 50 mg of powder in 1.7 mL of 80% (*v/v*) methanol acidified with HCl (approximately 1 mL/L), following a procedure analogous to the preparation of powder extracts for the antiglycation assays (Section 2.16)—i.e., vortexing, ultrasonication, refrigerated standing, further sonication and agitation, followed by centrifugation—but using the acidified methanol as the extraction solvent instead of the phosphate buffer, with sodium azide used for the antiglycation extracts.

#### 2.13.1. Folin–Ciocalteu Method

Total phenolics content was determined by the Folin–Ciocalteu colorimetric method of Siriwoharn et al. (2004) [61], as modified by Horszwald and Andlauer (2011) [62]. A microplate well was filled with 25 μL of sample and 250 μL of Folin–Ciocalteu reagent diluted with distilled water (1:15, *v/v*). After 10 min of incubation at room temperature, 25 μL of 10% (*w/v*) Na_2_CO_3_ was added, and the plate was kept in the dark for a further 20 min before the absorbance was read at 750 nm. A blank was prepared by replacing the sample with the corresponding solvent. Results were expressed as g gallic acid equivalents (GAE) per 100 g dry matter (DM). The Folin–Ciocalteu response is not specific to phenolic compounds because other reducing substances present in the extract can also reduce the reagent and contribute to the measured absorbance [63].

#### 2.13.2. Fast Blue BB Method

Total phenolics content was additionally determined using the Fast Blue BB reagent according to the protocol of Medina (2011) [64] with gallic acid (0–500 μg/mL) as the calibration standard and distilled water as the solvent for all reagents. In a microtube, 1 mL of appropriately diluted sample or standard solution was combined with 100 μL of 0.1% (*w/v*) Fast Blue BB reagent and mixed thoroughly for 1 min, after which 100 μL of 5% (*w/v*) NaOH solution was added. The reaction was left at room temperature in the dark for 90 min, and 200 μL aliquots were transferred to a microtiter plate for absorbance measurement at 420 nm. Results were expressed as g GAE per 100 g DM. This method was applied alongside the Folin–Ciocalteu method to the final (extracted) powder samples.

### 2.14. Antioxidant Capacity—TEAC ABTS Assay

Trolox equivalent antioxidant capacity (TEAC) was determined using the ABTS radical cation decolorization assay following the method of Re et al. (1999) [65], as modified by Silva et al. (2007) [66]. The ABTS^•+^ radical was generated by oxidizing 10 mL of 7 mM ABTS with 0.5 mL of 51.4 mM potassium peroxodisulfate and keeping the mixture in the dark for 16 h at room temperature. The stock solution was diluted with distilled water to an absorbance of 0.75 ± 0.05 at 734 nm. Aliquots of 10 μL of appropriately diluted sample were mixed with 290 μL of the ABTS^•+^ working solution, and the absorbance was read at 734 nm after 6 min in the dark using a Synergy H1 spectrophotometer (BioTek Instruments Inc., Santa Clara, CA, USA). Four replicates (*n* = 4) were analyzed per sample, and results were expressed as mmol Trolox equivalents (TE) per 100 g DM.

### 2.15. Antioxidant Capacity—FRAP Assay

The ferric-reducing antioxidant power (FRAP) assay was performed according to Benzie and Strain (1996) [67]. The FRAP reagent was prepared by mixing acetate buffer (300 mM, pH 3.6), 10 mM 2,4,6-tris(2-pyridyl)-s-triazine dissolved in 40 mM HCl, and 20 mM FeCl_3_ · 6H_2_O, in a ratio of 10:1:1 (*v/v/v*). To each well, 10 μL of diluted sample, 10 μL of distilled water, and 200 μL of FRAP reagent were added and mixed thoroughly. After 10 min, the absorbance was measured at 593 nm in four replicates (*n* = 4). Results were expressed as mmol TE per 100 g DM.

### 2.16. In Vitro Antiglycation Activity

Antiglycation activity was evaluated using four in vitro glycation models: BSA–glucose (BSA–Glu), BSA–fructose (BSA–Fru), BSA–methylglyoxal (BSA–MGO), and methylglyoxal–L-arginine (MGO–Arg). The procedures were adapted from Wang et al. (2011) [68] and Michalska-Ciechanowska et al. (2024) [22], with modifications to the extraction, dilution, reaction volumes, and incubation procedure described below.

For each powder formulation, duplicate portions with target masses of 10 and 50 mg were weighed into separate microcentrifuge tubes, resulting in two independently prepared extracts at each preparation level. Each portion was extracted with 1.7 mL of sodium phosphate buffer (50 mM, pH 7.4) containing 0.02% (*w/v*) sodium azide. The samples were vortexed, sonicated in an ultrasonic bath (Bandelin electronic GmbH & Co., Berlin, Germany) for 15 min, and stored at 4–5 °C for 20 h. They were then vortexed again, sonicated for a further 15 min, and centrifuged at 14,000 rpm for 7 min. The resulting supernatants were diluted 2.5-fold with the same buffer and used in the antiglycation assays.

The final reaction mixture contained 0.78 mg powder equivalents/mL when the extract was prepared from 10 mg of powder and 3.92 mg/mL when 50 mg were used. These concentrations were calculated from the initially weighed powder mass.

For each model, 0.5 mL of diluted powder extract was combined with 0.5 mL of 1.5 M glucose (BSA–Glu), 1.5 M fructose (BSA–Fru), or 60 mM methylglyoxal (BSA–MGO and MGO–Arg). After incubation at 37 °C for 2 h with shaking at 110 rpm (Incu-Shaker Mini, Benchmark Scientific, Sayreville, NJ, USA), 0.5 mL of BSA solution (30 mg/mL) was added to the BSA-based models, while 0.5 mL of L-arginine solution (60 mM) was added to the MGO–Arg model. The final 1.5 mL reaction mixtures contained 0.50 M glucose or fructose, 20 mM methylglyoxal, 10 mg/mL BSA, or 20 mM L-arginine, as applicable. For the control reactions, the powder extract was replaced with 0.5 mL of phosphate buffer containing sodium azide (uninhibited control) or 0.5 mL of 30 mM aminoguanidine (positive control; final concentration 10 mM). All phosphate-buffered solutions contained 0.02% (*w/v*) sodium azide.

After the addition of BSA or L-arginine, the reaction mixtures were incubated at 37 °C with shaking at 110 rpm and protected from light. Fluorescence measurements were initiated after 163 h of incubation. Immediately before measurement, a 250 µL aliquot of each reaction mixture was transferred to a black 96-well microplate. Fluorescence was measured using a Synergy H1 spectrophotometer (BioTek Instruments Inc., Santa Clara, CA, USA; read height 9.75 mm) at excitation/emission wavelengths of 340/420 nm for the BSA–Glu and BSA–Fru models, and 340/380 nm for the BSA–MGO and MGO–Arg models. In all four models, AGE inhibition (%) was calculated as:

(3) $$\mathrm{AGE inhibition}\left(\%\right)=\left(1-\frac{F_{\mathrm{sample}}}{F_{\mathrm{control}}}\right)\times 100,$$

where *F_sample_* is the fluorescence intensity of the reaction mixture containing the powder extract or aminoguanidine, and *F_control_* is the fluorescence intensity of the corresponding uninhibited reaction mixture containing phosphate buffer with sodium azide in place of the tested inhibitor.

### 2.17. Statistical Analysis

Statistical analyses were performed using Statistica 13 (StatSoft, Prague, Czech Republic) and Microsoft Excel. Results are presented as mean ± standard deviation for the stated number of replicates. Differences between formulations were evaluated by analysis of variance (ANOVA) followed by a Tukey’s HSD test. Pearson correlation coefficients were calculated to assess relationships between total phenolics content and antioxidant capacity (TEAC ABTS and FRAP).

## 3. Results and Discussion

### 3.1. Preliminary Selection of Aqueous Extraction Conditions

Preliminary trials were conducted to select a common herb/water ratio and extraction time for all herbal extracts. At a fixed temperature of 90 °C, total phenolics content (TPC) generally increased most rapidly during the first 15–30 min, followed by a slower increase, plateau, or decrease, depending on the botanical material and herb/water ratio (Figure A1). The extraction profiles were strongly matrix-dependent. Specifically, AV exhibited a continuous TPC increase during prolonged extraction under several conditions. In contrast, MP, UD, and both binary mixtures generally showed limited additional increases or lower TPC values at longer extraction times.

The effect of the herb/water ratio was likewise matrix-dependent. When TPC was expressed relative to the dry herbal material, increasing the ratio from 1 to 2 g/100 g water resulted in consistently higher TPC values for AV and higher values for MP at the longest extraction time but did not produce the same response for UD or the binary mixtures. A further increase to 3 g/100 g water did not consistently increase TPC. For example, after 60 min, the TPC of AV was higher at 2 g/100 g water than at 3 g/100 g water. Because the preliminary trials included only two independent extractions, these observations were evaluated descriptively without inferential statistical analysis.

Based on the overall extraction profiles, an herb/water ratio of 2 g/100 g water and an extraction time of 60 min at 90 °C were selected as common conditions for preparing the extracts subsequently used for powder production. These conditions worked reasonably well across all three single herbs and both binary mixtures rather than corresponding to the individually optimized conditions for each material. The extraction time of 60 min covered the main phase of TPC increase for MP and UD while allowing substantial phenolic extraction from the more gradually responding AV matrix. Nedyalkov et al. (2015) likewise found that the extraction rate from *A. mollis* was highest during the first hour, although their use of 20% aqueous ethanol and a related *Alchemilla* species limits direct comparison [69]. Goldsmith et al. (2014) reported an optimum of 90 °C and 70 min for aqueous phenolic extraction from olive leaves, providing an additional methodological point of comparison despite the different botanical matrix [70]. After reaching their highest measured values, TPC declined at longer extraction times in MP, UD, and both binary mixtures, whereas it continued to increase in AV throughout the 120 min period (Figure A1). One possible interpretation is that, in MP, UD, and the binary mixtures, continued phenolic release no longer compensated for changes occurring during prolonged heating. Thermal transformations of extractable phenolics and matrix-dependent differences in their release or stability have been reported in other plant systems [71]. Oxygen availability has also been shown to affect phenolic stability during heating in apple puree [72]. However, because individual phenolic compounds, transformation products, and extraction kinetics were not assessed, the present data cannot distinguish these mechanisms from assay-related variability. The continued increase in AV TPC should therefore not be interpreted as evidence that phenolic extraction remained incomplete after 120 min.

Key findings:

• TPC extraction profiles were strongly matrix-dependent, with AV showing a more sustained increase than MP, UD, and the two binary mixtures.
• An herb/water ratio of 2 g/100 g water and 60 min at 90 °C was selected as a standardized compromise for all five botanical systems rather than as a matrix-specific optimum.

### 3.2. Physical and Process Properties

Before carrier addition, the AV + UD extract appeared visibly more turbid than the other four aqueous extracts; however, turbidity was not quantified. The refractometric soluble-solids readings of the feed formulations varied primarily according to carrier formulation rather than botanical material (Table 1). Carrier-free feeds ranged from 0.55 to 0.65 °Brix, whereas feeds containing inulin (IN) or maltodextrin (MD) ranged from 5.30 to 5.65 °Brix. The two carriers, therefore, produced broadly similar increases. The IN + PPI formulations consistently showed lower readings of 4.40–4.60 °Brix. This difference may partly reflect the limited and formulation-dependent aqueous solubility of PPI, which is influenced by its globular structure and aggregation state [73]. Moreover, soluble proteins contribute to refractive index in a composition-dependent manner [74]. Physical mixing of PPI and inulin produced only minor changes in particle size and protein secondary structure [75]. Because °Brix is expressed as a sucrose-equivalent concentration, readings obtained for these complex formulations should be regarded as operational refractometric estimates instead of direct gravimetric measurements of total solids. Nevertheless, because PPI solubility and gravimetric total solids were not determined independently, the lower readings of the IN + PPI formulations cannot be attributed conclusively to either limited protein dissolution or differences in refractive properties.

Spray drying outlet temperatures varied within a narrow range of 94–98 °C. Among the carrier-containing formulations, MD feeds reached the highest outlet temperatures across all five botanical systems. Spray drying produced powders with lower moisture content than freeze-drying (Table 2). The moisture content of the spray-dried powders ranged from 1.13 to 4.18%, compared with 4.32–10.49% for the freeze-dried products. The largest differences were observed in the carrier-free formulations, where FD had three to nine times the moisture content. Carrier addition markedly reduced the gap between spray- and freeze-dried moisture content relative to the carrier-free formulations, where freeze-dried powders contained 3.4 to 9.3 times more moisture than their spray-dried counterparts. Among the carrier-containing formulations, this ratio ranged from 1.6- to 3.5-fold for maltodextrin, 1.5- to 2.4-fold for inulin, and 2.0- to 2.6-fold for the inulin–PPI blend.

Processing behavior also differed qualitatively among formulations. Carrier-free (C) powders, particularly those produced by FD, showed a greater apparent stickiness during the manual grinding and transfer into storage bags, which may have caused some material loss during powder recovery. Carrier-free spray-dried extracts also showed visible adhesion to the cyclone. Wall deposition can reduce gravimetric powder recovery because deposited material does not reach the collection vessel [76,77]. In spray-dried plant extracts, such deposition may partly involve glass-transition-related stickiness. In a comparative study of seven medicinal-plant extracts, materials containing reported sugars exhibited relatively low glass transition temperatures (*T_g_*); however, a low *T_g_* was not consistently accompanied by a low process yield [78]. Relatively high-*T_g_* carriers, including maltodextrin and inulin, are used during SD of sugar-rich products to increase formulation *T_g_* and mitigate stickiness; however, their performance depends on carrier type, concentration, and product matrix [79]. Because neither *T_g_* nor the concentrations of individual low-molecular-weight sugars were determined in the present study, this mechanism remains a plausible explanation rather than a demonstrated cause.

Feed volume differed between SD formulations: carrier-free extracts were processed in 300 mL batches, whereas carrier-containing formulations were processed in 100 mL batches. Because feed volume and carrier addition were not varied independently, their respective contributions to SD recovery cannot be separated. Carrier-containing formulations were nevertheless easier to handle during subsequent weighing and processing and showed descriptively higher SD recovery than the corresponding carrier-free formulations in four of the five botanical systems, with AV + UD as the exception. No corresponding consistent relationship between carrier addition and recovery was observed after FD.

A similar carrier-dependent difference between drying techniques was reported for cranberry juice powders: FD products contained approximately 2.5–4.1 times more moisture than SD products with inulin and 8.2–12.5 times more with maltodextrin [80]. Higher moisture contents after FD than after SD were likewise reported for *Echinacea purpurea* root extract powders containing maltodextrin, PPI, or their blend [28]. Kułaga et al. (2025) suggested that the lower moisture content of SD powders could reduce their susceptibility to caking [28]. Because caking and storage behavior were not assessed in the present study, this possible technological advantage of SD remains to be verified. Nevertheless, the drying technique alone does not determine the residual moisture content. However, this pattern is not universal. Kucharska-Guzik et al. (2025) found no significant differences in moisture content between SD and FD *Cistus creticus* extract powders, with moisture contents ranging from 3.42% to 4.36% [24]. The lower moisture contents obtained by spray drying in the present study and some previous studies can be related to feed atomization: the resulting small droplets provide a large surface area for rapid heat and mass transfer during contact with hot air [28,81]. In contrast, freeze-drying involves ice sublimation during primary drying, followed by the desorption of residual sorbed water during secondary drying. The higher moisture contents of the freeze-dried powders may therefore be associated with less extensive removal of sorbed water during secondary drying under the conditions applied [23,28].

Water activity ranged from 0.116 to 0.366 in the spray-dried powders and from 0.118 to 0.498 in the freeze-dried powders. All measured values were below 0.50 and below the reported lower water-activity limit for microbial cell division [82]. Although these values suggest conditions unfavorable for microbial proliferation, low-water-activity foods are not necessarily microbiologically sterile because viable microorganisms may persist even when growth is prevented [83]. Therefore, water activity alone cannot establish the microbiological safety or storage stability of a specific food powder. Neither property was evaluated directly in the present study.

Carrier addition was associated with lower water activity in most formulations. The carrier-free powder had the highest *a_w_* in all five freeze-dried botanical systems and in four of the five spray-dried systems. This pattern is consistent with previous studies. Zokti et al. (2016) reported lower *a_w_* for spray-dried green tea extract with MD (0.28) than for the non-encapsulated control (0.45) [84]. Powders prepared from aqueous extracts of *Ginkgo biloba* L. and *Clitoria ternatea* L. with 10% IN or MD likewise had lower *a_w_* than the corresponding carrier-free controls for both drying techniques [85].

The relative performance of IN and MD depended on the drying technique applied. MD gave a lower *a_w_* than IN in four of the five spray-dried botanical systems, whereas IN gave a lower value in four of the five freeze-dried systems. A similar dependence of carrier performance on the drying method was reported for cranberry juice powders [80]. The IN + PPI formulations generally also had low water activity, with the lowest values recorded for UD after spray drying and freeze drying (0.116 and 0.118, respectively). This result cannot be attributed specifically to PPI because no formulation containing PPI alone was examined. Low *a_w_* values of 0.16–0.22 were also reported for spray-dried aqueous *Cyani flos* extract powders prepared with inulin or inulin–pectin carriers [86]. Only one inulin–PPI ratio (95:5, *w/w*) was evaluated; therefore, the present results do not identify an optimal carrier composition. Because PPI concentration and formulation can influence feed rheology and atomization during spray drying [87,88], future studies should examine a broader range of PPI proportions and determine their effects on drying performance and powder properties.

Variations in water activity may also be caused by the drying process itself. Brzezowska et al. (2024) reported the highest average *a_w_* for freeze-dried cranberry pomace extract powders and the lowest after vacuum drying at 90 °C [89]. Their study compared freeze-drying with vacuum drying rather than spray drying, so it provides contextual rather than directly comparable evidence.

Spray process efficiency ranged from 47.2 to 82.0%. Carrier addition markedly increased process efficiency for AV, MP, and AV + MP, whereas the response was less pronounced for UD and absent for AV + UD, whose carrier-free formulation already showed a process efficiency of 81.9%. The observed range was comparable to the 64.63–87.23% reported for spray-dried nettle leaf extracts by Cegledi et al. (2022) [51]. Cam et al. (2020) similarly reported that adding maltodextrin to an aqueous peppermint extract obtained by pressurized liquid extraction significantly increased the spray drying yield to 64.4% compared with the carrier-free formulation (*p* < 0.05) [90]. The higher process efficiency of carrier-containing formulations may reflect improved powder recovery due to reduced stickiness and wall deposition during spray drying, although these mechanisms were not measured directly. Freeze-drying process efficiency was higher overall (74.8–98.1%), primarily because the product could be recovered directly from the drying vessel, whereas spray drying involved additional material losses in the drying chamber and cyclone. Consequently, the higher gravimetric process efficiency after freeze-drying should not be interpreted as evidence of greater overall process or energy efficiency.

Previous studies produced *M. piperita* powders from concentrated aqueous extracts obtained before or after hydrodistillation [45], peppermint tea extract using an experimental hot-bed spray dryer [46], hot-water extracts with maltodextrin [47], peppermint hydrosol spray-dried with maltodextrin [48], pressurized hot-water extracts [49], and freeze-dried infusions prepared from peppermint alone and from a 1:1 green tea–peppermint mixture [50]. For *U. dioica*, Mousavi Kalajahi and Ghandiha (2022) spray-dried a 24 h aqueous extract with maltodextrin [52], whereas Cegledi et al. (2022) used microwave-assisted extraction with 30% aqueous ethanol and evaluated maltodextrin or β-cyclodextrin, alone or combined with gum arabic [51]. Because these studies differed in extraction method, feed composition, carrier formulation, drying equipment, and yield calculation, their recovery values are not directly comparable with those obtained in this research paper. The present recovery values should therefore be interpreted as specific to the procedures used rather than as an intrinsic ranking of drying methods or carriers.

Carrier addition consistently increased powder lightness (Table 3). All carrier-containing powders had higher L* values than their carrier-free counterparts after both drying methods, although the size of the increase differed among formulations. The largest difference occurred within the freeze-dried AV + UD series, where L* increased from 32.08 without a carrier to 79.04 with IN. These values were also the minimum and maximum recorded in the entire dataset. When the two drying methods were examined separately, moisture content and L* followed opposite trends. Higher moisture coincided with lower L* among the freeze-dried powders (*r* = −0.876), whereas the spray-dried powders showed a positive correlation (*r* = 0.760). Because carrier composition varied across the same formulations and was related to both measurements, these coefficients describe the pattern within the present dataset but do not isolate a direct effect of moisture on powder color. The comparatively higher variability observed in the color parameters of carrier-free control powders, especially under freeze-drying, may partly reflect caking and the lower overall powder mass recovered for these formulations, which limited the extent to which the sample surface covered the measurement area during colorimetric reading. Studies of cranberry juice and chokeberry pomace extract powders have likewise reported higher L* values after the addition of IN or MD in several formulations. The degree of lightening varied between carriers and drying treatments [80,91].

Variation along the green–red a* axis was most pronounced in the MP and UD formulations. Freeze-dried MP–C reached the highest value (9.07), whereas the carrier-containing UD powders were the only formulations that showed negative a* values. For each of these UD formulations, freeze-dried powders had lower a* values than their spray-dried counterparts, with the lowest value (−3.53) measured for freeze-dried UD–IN + PPI. Freeze-dried nettle leaf powders have previously been reported to retain greener coloration and higher chlorophyll contents than convectively dried powders [92]. The direction of the change observed in the present UD powders is consistent with that finding, although chlorophyll retention cannot be established from a* alone because the individual pigments were not quantified.

All powders had positive b* values, indicating a shift toward yellow, with values ranging from 16.75 for spray-dried UD–MD to 30.79 for freeze-dried AV–C. Comparison of IN and MD showed a reversal between the two drying techniques. Among the spray-dried powders, the MD formulations had numerically lower b* values than the corresponding IN formulations in all five botanical systems and lower a* values in four systems. After freeze-drying, MD had higher a* values than IN in all five systems and higher b* values in four. The relative color of the IN and MD formulations thus changed with the drying method; neither carrier consistently produced a greener, redder, or yellower powder. A similar reversal was observed for *Cistus creticus* extract powders, where the relative L*, a*, and b* values of the IN and MD formulations changed between spray drying and freeze-drying [24]. Carrier-related color differences across drying processes were also reported for cranberry juice powders [80].

The browning index ranged from 24.11 to 111.99 (Figure 1). Within every botanical system, the carrier-free formulation had the highest BI after both drying methods. Carrier-free powders ranged from 72.46 to 97.76 after spray drying and from 90.67 to 111.99 after freeze-drying. The corresponding ranges for the carrier-containing powders were 24.11–48.64 and 29.49–59.79, respectively. Their lower BI values mainly reflected higher L* and generally lower a* values, showing that the carrier matrix markedly changed the visible color of the powders.

The relative ranking of IN and MD by BI also changed with the drying method. Among the spray-dried powders, MD had a numerically lower BI than IN in four of the five botanical systems. Sea buckthorn juice powders showed a qualitatively similar trend, with less pronounced browning in MD formulations than in those containing IN [30]. However, Tkacz et al. (2020) measured browning spectrophotometrically in powder extracts, whereas BI in the present study was calculated from CIE L*a*b* coordinates. The absolute values obtained using the two approaches are therefore not directly comparable. After freeze-drying, MD had a higher BI than IN in four of the five botanical systems. Freeze-drying also produced higher BI values for four of the five carrier-free powders and for all MD formulations, but lower values for four of the five formulations containing IN or IN + PPI. Thus, neither drying method produced consistently higher BI across all carrier groups.

In this study, BI was derived from CIE L*a*b* values and describes the brown appearance of the powders rather than a specific chemical reaction. Native plant pigments and carrier addition also affected the measured color. A higher BI therefore cannot by itself be attributed to Maillard reactions, caramelization, or pigment degradation.

Key findings:

• SD produced powders with lower moisture contents, whereas FD resulted in higher overall process efficiency; therefore, neither drying method was superior across all evaluated physical and process properties.
• Carrier-containing formulations generally had lower water activity, were easier to handle, and showed numerically higher SD process efficiency in four of the five botanical systems. However, the effects of carrier addition and feed volume on SD process efficiency could not be separated, and no consistent carrier-related pattern was observed after FD.
• Carrier-containing powders were lighter and had lower calculated BI values than the corresponding carrier-free powders in every botanical system after both drying methods; however, BI describes visible powder color and does not directly measure chemical browning.

### 3.3. Phenolics Content and Antioxidant Capacity

Table 4 presents the total phenolics content and antioxidant capacity measured in methanolic extracts of the spray- and freeze-dried powders. All results are expressed per 100 g DM of the final powder. In carrier-containing formulations, this mass includes both herbal extract solids and the added carrier.

Carrier addition markedly affected the values reported in Table 4. Carrier-free spray-dried powders had Folin–Ciocalteu TPC values of 4.12–28.65 g GAE/100 g DM, while carrier-free freeze-dried powders gave values of 3.31–36.99 g GAE/100 g DM. After carrier addition, the respective ranges fell to 0.77–3.62 and 0.63–3.66 g GAE/100 g DM. Part of the dry mass of these powders consisted of the carrier rather than the herbal extract. Their lower TPC values thus partly reflect dilution and do not, on their own, show that phenolic compounds were degraded during drying. Cegledi et al. (2022) found the same general trend in spray-dried nettle extracts, where higher carrier proportions resulted in lower powder loading capacity [51]. Among the carrier-containing powders, IN generally gave higher ABTS and FRAP values than MD, and several of these differences were significant according to Tukey’s HSD test. In contrast, the Folin–Ciocalteu TPC values of the IN and MD formulations did not differ significantly within any botanical system after either drying technique.

AV had the highest values in every assay after both drying methods. In most comparisons, the carrier-free powders ranked in the order AV > AV + MP > MP > AV + UD > UD. The only exception was the Fast Blue BB assay after spray drying, in which MP gave a slightly higher response than AV + MP. AV + UD consistently remained between AV and UD, while AV + MP remained between its constituent herbs except in this single comparison. The generally higher values of the mixtures relative to MP or UD alone reflect the contribution of the phenolic-rich AV component. They do not establish synergy between the herbs. No additive reference or concentration–response interaction analysis was included in the present study. Kurin et al. (2025), for example, evaluated antioxidant interactions in green tea–peppermint combinations using concentration–response data and a combination index [50]. Synergistic or antagonistic effects were not assessed here.

The carrier-free AV powders contained 28.65 g GAE/100 g DM after spray drying and 36.99 g GAE/100 g DM after freeze-drying. These values were numerically higher than the 23.73 g GAE/100 g extract reported by Pekacar et al. (2026) [93] for a freeze-dried aqueous preparation of *A. vulgaris*. However, direct comparison is limited by differences in extraction procedures and by the unclear sample normalization in that study.

For peppermint, Cam et al. (2020) reported 6.3 ± 0.3 g GAE/100 g powder for SD pressurized-water extract formulated with equal amounts of maltodextrin and gum arabic [90]. Gum arabic was added especially to preserve the flavor of peppermint. This value was lower than that of the SD carrier-free MP powders in the present study (15.41 g GAE/100 g DM) but higher than that of the maltodextrin formulations (1.75 g GAE/100 g DM). The difference may partly reflect their more concentrated starting extract, diluted to 6 g/100 g soluble solids before carrier addition, well above the 0.65 g/100 g used in the present study (Table A1). Berktas and Cam (2021) additionally reported 10.77–11.07 g GAE/100 g powder for maltodextrin-containing spray-dried peppermint hydrosols obtained as by-products of hydrodistillation [48]; however, the different feed material and formulation basis limit direct comparison with the present aqueous-extract powders.

The carrier-free UD powders contained 33.1–41.2 mg GAE/g DM. Mousavi Kalajahi and Ghandiha (2022) reported 18.19–56.67 mg GAE/g powder for spray-dried aqueous nettle extracts containing maltodextrin [52]. The carrier-free UD results are within this published range, while the carrier-containing UD powders in the present study contained only 6.3–8.4 mg GAE/g DM. Differences in the herbal material, extraction conditions, carrier proportion, and composition of the final powder prevent a direct comparison of phenolic retention.

AV and MP did not give the same ratio between the two phenolic assays. Folin–Ciocalteu gave the higher value for every AV powder, while Fast Blue BB gave the higher value for every MP powder. The chemistry of the assays offers a possible explanation. Color development in the Folin–Ciocalteu assay follows reduction of the reagent, and nonphenolic reducing compounds can contribute to the measured response [63]. Fast Blue BB reacts with phenolic groups under alkaline conditions to form colored azo products. Lester et al. (2012) found that Fast Blue BB did not interact with ascorbic acid or sugars in strawberry extracts [94]. Zugazua-Ganado et al. (2024) likewise observed substantially less interference from ascorbic acid and fructose with Fast Blue BB than with Folin–Ciocalteu in tea infusions and fruit drinks [95]. These differences in assay selectivity may partly explain why AV and MP gave different relative responses with the two methods.

The comparison between spray drying and freeze-drying varied with the herbal material, carrier, and phenolic assay. Carrier-free AV had a Folin–Ciocalteu TPC of 28.65 g GAE/100 g DM after spray drying and 36.99 g GAE/100 g DM after freeze-drying. MP gave similar values with both methods, while freeze-dried UD and binary-mixture powders had numerically lower TPC than their spray-dried counterparts. Differences among the carrier-containing formulations were generally smaller, and Fast Blue BB gave no consistent ranking of the two drying methods. Kucharska-Guzik et al. (2025) also found that the carrier altered the comparison in *Cistus creticus* powders [24]. Folin–Ciocalteu values were nearly identical after spray drying and freeze-drying with inulin (5.07 and 5.12 g GAE/100 g DM), while the corresponding maltodextrin powders gave 4.58 and 2.93 g GAE/100 g DM.

TEAC ABTS and FRAP did not respond to drying in the same way. Every freeze-dried formulation gave a higher TEAC ABTS value than its spray-dried counterpart. Among the carrier-free powders, TEAC ABTS activity ranged from 13.78 to 228.58 mmol TE/100 g DM after spray drying and from 20.22 to 307.47 mmol TE/100 g DM after freeze-drying. FRAP moved in the opposite direction in 16 of the 20 paired formulations; the exceptions were the MD-containing AV, MP, UD, and AV + MP powders. The higher TEAC ABTS values alone do not indicate a general increase in antioxidant capacity after freeze-drying.

The FRAP assay measures the reduction of the Fe(III)–TPTZ complex at acidic pH [63,96]. This difference in reaction chemistry is consistent with the higher TEAC ABTS but mostly lower FRAP values measured for the freeze-dried powders.

For methanolic extracts prepared from the 20 powder formulations obtained by each drying, Folin–Ciocalteu TPC correlated strongly with Fast Blue BB values (SD: *r* = 0.969; FD: *r* = 0.949), with TEAC ABTS values (SD: *r* = 0.945; FD: *r* = 0.997), and with FRAP values (SD: *r* = 0.955; FD: *r* = 0.997). This covariation is consistent with methanol-extractable phenolic constituents contributing to the antioxidant capacity measured under the applied assay conditions. However, carrier-free powders generally gave high values in all four assays, whereas carrier-containing powders clustered at lower values. Because the correlations were calculated across both groups, this separation may partly account for the high coefficients. The analysis therefore demonstrates parallel variation among the assay results at the formulation level; determining the contributions of individual phenolic compounds would require compositional analysis of the extracts.

Key findings:

• Carrier-free powders had the highest TPC and antioxidant capacity on a final-powder dry-mass basis, whereas the lower values of carrier-containing powders partly reflected dilution of the herbal solids by the carriers.
• AV gave the highest values in all phenolic and antioxidant assays after both drying methods. The binary mixtures generally showed intermediate responses, and the results did not establish synergistic interactions between their constituent herbs.
• TPC correlated strongly with Fast Blue BB, TEAC ABTS, and FRAP within both drying methods, although TEAC ABTS and FRAP differed in their responses to SD and FD; neither drying method was consistently superior across all assays and formulations.

### 3.4. Antiglycation Activity

Figure 2, Figure 3, Figure 4 and Figure 5 show the inhibition of fluorescent AGE formation by extracts prepared from 10 mg and 50 mg portions of the powders, corresponding to final nominal concentrations of 0.78 and 3.92 mg powder equivalents/mL in the reaction mixtures. All powder extracts showed positive apparent inhibition of fluorescent AGE formation across the four glycation models. Because extract-only fluorescence blanks were not measured, a contribution from intrinsic extract fluorescence or other optical interference cannot be ruled out. Aminoguanidine was included in every assay as the positive control at a final concentration of 10 mM; its hydrazine group reacts with reactive dicarbonyl intermediates, particularly methylglyoxal and glyoxal, forming triazine adducts and thereby reducing their availability for protein modification and subsequent AGE formation [97,98]. Against this reference, the herbal powder extracts showed considerable variation among models, herbal matrices, and formulations, as detailed below.

In the BSA–Glu model (Figure 2), mean apparent inhibition increased from 39.8% at 0.78 mg powder equivalents/mL to 45.5% at 3.92 mg powder equivalents/mL across all formulations and drying techniques. Carrier-free MP powders gave the highest values, reaching 79.9% and 86.2% after spray drying and 78.3% and 85.7% after freeze-drying at the lower and higher concentrations, respectively. Carrier-containing UD powders showed the lowest activity; for example, apparent inhibition by freeze-dried UD–MD decreased from 13.7% to 7.8% as the concentration increased. The same direction of change was observed for every carrier-containing UD and AV + UD formulation after both drying treatments. The response in this model was thus not uniformly concentration-dependent. Because extract-only fluorescence blanks were not measured, a contribution from intrinsic extract fluorescence or other optical interference cannot be ruled out.

In the BSA–Fru model (Figure 3), mean apparent inhibition increased from 54.0% at 0.78 mg powder equivalents/mL to 66.9% at 3.92 mg powder equivalents/mL. At the higher concentration, spray-dried MP–C gave the highest value (88.2%), whereas freeze-dried UD–MD gave the lowest (34.9%). When all botanical systems, concentrations, and drying methods were considered together, carrier-free powders averaged 73.1% inhibition, compared with 56.2% for the carrier-containing formulations. The corresponding means for spray- and freeze-dried powders were 63.8% and 57.2%, respectively. This numerical difference of 6.6 percentage points was larger than the average difference between the two drying methods in the other glycation models.

The generally higher inhibition percentages in the BSA–Fru model than in the BSA–Glu model may be related to differences in the glycation reactions initiated by the two sugars. Fructose glycates proteins more rapidly than glucose, partly because a larger proportion of fructose occurs in the reactive open-chain form [99,100]. The BSA–Fru results should be considered separately from those obtained in the BSA–Glu model. In a kinetic study of BSA glycoxidation, Sadowska-Bartosz et al. (2014) measured a 2.7-fold higher initial rate of fluorescent glycation-product formation with fructose than with glucose and found that the effects of individual inhibitors varied with the glycating sugar [100]. The higher inhibition percentages obtained in the BSA–Fru model should thus be regarded as responses specific to fructose-mediated glycation rather than as evidence of greater antiglycation activity in general. Moreover, because inhibition was calculated against a separate uninhibited control for each model, the percentages do not directly compare the absolute amounts of AGEs formed with glucose and fructose.

In the BSA–MGO model (Figure 4), increasing the powder-equivalent concentration raised the mean apparent inhibition from 41.1% to 68.8%. Carrier-free AV powders gave values of 85.9–86.2% at the lower concentration and 97.4–97.5% at the higher concentration, depending on the drying method. Carrier-free AV + MP and MP powders similarly reached approximately 97% inhibition at 3.92 mg powder equivalents/mL. The lowest responses were obtained for carrier-containing UD powders: freeze-dried UD–MD gave 17.0% inhibition at the lower concentration, whereas freeze-dried UD–IN gave 37.0% at the higher concentration. When all botanical systems, concentrations, and drying methods were considered together, carrier-free powders averaged 83.4% inhibition, compared with 45.5% for formulations containing IN, MD, or IN + PPI.

The BSA–MGO model bypasses the initial reaction between a reducing sugar and the protein and instead examines inhibition of MGO-mediated glycation, representing an intermediate stage of the process [68]. The high apparent inhibition obtained for carrier-free AV and AV + MP shows that their buffer-extractable constituents remained effective in this MGO-initiated system. It does not, however, establish MGO trapping as the underlying mechanism. Wang et al. (2011) supported carbonyl scavenging by quantifying MGO depletion by HPLC and tentatively identifying catechin–MGO and catechin–glyoxal adducts using HPLC–ESI–MSn [68]. Neither MGO depletion nor the formation of corresponding adducts was measured in the present study. For MP specifically, direct evidence of MGO trapping is available at the compound level: eriocitrin and eriodictyol, the major peppermint flavonoids, were shown to form mono-adducts with MGO, whereas rosmarinic acid did not, despite showing intermediate inhibitory activity in isolation, suggesting a contribution from mechanisms other than direct carbonyl trapping [9]. Rosmarinic acid has also been reported to inhibit MGO-induced AGE formation in human serum albumin in vitro [101]. Eriocitrin’s disproportionately large contribution to the overall antiglycation activity of a peppermint extract relative to its individual potency further illustrates that whole-extract performance cannot be inferred from single-compound activity alone [9].

The strongest concentration response was observed in the MGO–Arg model (Figure 5), where mean apparent inhibition increased from 57.0% at 0.78 mg powder equivalents/mL to 87.4% at 3.92 mg powder equivalents/mL. Carrier-free powders (C) showed a mean inhibition of 92.6% at the lower concentration, increasing to 98.6% at the higher concentration. Individual carrier-free formulations reached 97.3–99.3% at 3.92 mg powder equivalents/mL, with the highest value recorded for spray-dried AV + MP–C (99.3%). Even the lowest value at the higher concentration, obtained for spray-dried UD–MD, was 69.8%. The MGO–Arg model describes the reaction between MGO and the guanidino group of arginine, which can produce the fluorescent AGE argpyrimidine [68]. The measured endpoint was limited to fluorescent AGE formation in the MGO–Arg reaction and provides no direct information about other AGE-forming pathways. At 3.92 mg powder equivalents/mL, the carrier-free formulations clustered within a narrow range of 97.3–99.3%. Ranking these samples based on such small numerical differences would not be meaningful.

Across the four models, differences associated with carrier presence were more pronounced than those associated with drying technique. The difference between the mean apparent inhibition of carrier-free and carrier-containing powders ranged from 16.9 percentage points in BSA–Fru to 37.9 percentage points in BSA–MGO. By comparison, the difference between spray and freeze-dried powders ranged from 1.2 to 6.6 percentage points. Because the assays compared equal masses of final powder rather than equivalent masses of herbal extract solids, part of this difference reflects dilution of the botanical material by the carriers. Michalska-Ciechanowska et al. (2024) likewise found that carrier addition and carrier type had a more evident influence on the antiglycation capacity of haskap juice powders than on powders prepared from phenolic-enriched fractions obtained by treating the juice with XAD-16 resin [22]. Their results also show that the response to carrier addition depends on the composition of the dried matrix. In the present study, the possible effects of the carriers on the extraction or accessibility of active constituents cannot be separated from the dilution effect. Carrier-only controls were not analyzed in the phenolic, antioxidant, or antiglycation assays; consequently, a direct contribution of the carriers themselves to the measured responses cannot be excluded.

The pooled mean apparent inhibition was 51.6% for IN, 51.5% for MD, and 49.5% for IN + PPI. Thus, neither IN nor MD showed a recurring numerical advantage across the four models. These cross-model means were not used for statistical comparison, and the slightly lower IN + PPI value cannot be attributed to PPI, as no PPI-only formulation was tested.

The difference between spray drying and freeze-drying was relatively small in three of the four models. Across the paired formulations and both extract concentrations, the mean absolute difference was 2.04 percentage points in BSA–Glu, 1.36 percentage points in BSA–MGO, and 1.73 percentage points in MGO–Arg. The BSA–Fru model differed: spray-dried powders gave higher apparent inhibition in every paired comparison, with a mean difference of 6.62 percentage points. Thus, neither drying method was consistently superior across all four glycation models. Michalska-Ciechanowska et al. (2024) also found that comparisons between spray and freeze-dried haskap powders changed with the glycation model, carrier, and type of feed material [22].

When the four models and both concentrations were pooled, the highest descriptive means were obtained for AV + MP and MP (66.3% and 66.0%), followed by AV (63.0%), AV + UD (53.5%), and UD (39.0%). These pooled values conceal differences among the individual reaction systems. MP gave the strongest response in BSA–Glu, whereas AV was the most active botanical material in BSA–MGO. The binary mixtures were not consistently more active than either of their constituent herbs. Their results cannot establish synergy or antagonism because no expected additive response was calculated from the mixture proportions at an equivalent amount of botanical solids.

The antiglycation results only partly followed the TPC and antioxidant measurements. UD generally showed lower values in both datasets, and carrier-free powders generally gave high values in the chemical and antiglycation assays. However, AV had the highest TPC, while MP and AV + MP showed slightly higher apparent antiglycation inhibition when the four models were pooled. The TPC ranking therefore did not fully reproduce the antiglycation ranking. At the compound level, Piwowar et al. (2019) found contrasting concentration responses: quercetin inhibited AGE formation more strongly at 20 µM than at 2 µM, whereas catechin showed the opposite pattern [102]. These findings suggest that phenolic composition may contribute to the mismatch between TPC and antiglycation responses, although individual phenolic compounds were not quantified in the present powders. Wang et al. (2011) likewise observed substantial differences in the responses of berry extracts among glycation models and associated these differences with phenolic composition and the reaction chemistry represented by each model [68]. A pooled correlation across all four models would be difficult to interpret because it would combine different glycation reactions, extract concentrations, and levels of carrier dilution. Any subsequent correlation analysis should consequently be performed separately for each model and powder-equivalent concentration. The relationship between antioxidant capacity and antiglycation activity also appears to vary among botanical materials and assay systems. Positive correlations between inhibition of AGE formation and both ABTS and FRAP antioxidant capacity were reported across 18 tea infusions [58]. In contrast, Ramkissoon et al. (2012) found no significant correlation of antiglycation activity with either FRAP or DPPH antioxidant capacity across ten culinary herb and spice extracts, including peppermint [59].

The experiments assessed the inhibition of fluorescent product formation in accelerated in vitro glycation systems. Extracts were compared on a powder-equivalent basis rather than at known concentrations of dissolved active compounds. Extract-only blanks were not included, so interference from intrinsic fluorescence, fluorescence quenching, or inner-filter effects of the naturally colored extracts cannot be excluded. The high substrate concentrations were used to accelerate glycation over several days and do not reproduce physiological exposure conditions. Sadowska-Bartosz et al. (2014) similarly cautioned that results obtained with high sugar concentrations in accelerated BSA glycation models cannot be extrapolated directly to in vivo conditions [100]. The present results show apparent inhibition of fluorescent AGE formation in vitro, but they do not provide evidence of antidiabetic efficacy or other health effects in vivo. Apparent inhibition of fluorescent AGE formation was measured only in the resulting powders, not in the corresponding aqueous extracts or liquid feed formulations. Therefore, its retention during carrier addition and drying could not be quantified. Whether this response would persist during subsequent food processing or cooking was likewise not evaluated directly.

No single formulation performed best across all measured properties. Carrier-free powders had the highest TPC values on a dry-mass basis (4.12–28.65 g GAE/100 g DM for spray-dried, and 3.31–36.99 g GAE/100 g DM for freeze-dried formulations) and generally gave the highest antioxidant (TEAC ABTS up to 307.47 mmol TE/100 g DM) and antiglycation responses (mean apparent inhibition 58.5–95.6% across the four models, compared with 37.3–64.4% for carrier-containing formulations). However, the carrier-free powders also had higher water activity, with values up to 0.498, and were more difficult to recover during spray drying. The lowest recovery, 47.2%, was observed for the carrier-free AV formulation. Carrier addition improved powder recovery (e.g., from 47.2% to 76.9% for AV) and generally lowered water activity to as low as 0.116 while diluting the herbal content of the powder and reducing TPC to 0.63–3.66 g GAE/100 g DM. This dilution likely contributed to the lower functional activity measured on a powder-mass basis, although possible effects of the carriers on the extraction or accessibility of active constituents cannot be excluded. Among the carrier-containing formulations, IN frequently gave higher TEAC ABTS and FRAP values than MD, but this advantage was not observed in the antiglycation assays, where IN, MD, and IN + PPI gave nearly identical mean inhibition (51.6%, 51.5%, and 49.5%, respectively). Spray drying produced powders with lower moisture contents (as low as 1.13%) than freeze-drying (as low as 4.32%), whereas freeze-drying gave higher powder recovery and TEAC ABTS values without consistently increasing TPC, FRAP, or antiglycation activity. Formulation selection will depend on whether the main priority is powder recovery and low water activity or measured functional activity on a product-mass basis.

These trade-offs should also be considered in relation to potential food applications. A 2% addition of hydroethanolic peppermint extract to yogurt was previously reported to increase DPPH radical-scavenging activity by approximately 39% relative to the control while maintaining acceptable sensory properties during refrigerated storage [103]. Nettle-derived ingredients have been investigated in bakery, dairy, and meat products and in edible films, while the nutritional composition and food-functional properties of *U. dioica* have also been reviewed more broadly [104,105]. These studies provide an application precedent for ingredients derived from *M. piperita* and *U. dioica*. Nevertheless, differences in extraction solvent, composition, carrier content, dose, and food matrix prevent direct extrapolation to the aqueous powders investigated here. The performance of the prepared *A. vulgaris* powders in food matrices also remains to be established.

Inulin may provide additional formulation flexibility because it has been applied in dairy, meat, bakery, confectionery, plant-based, and gluten-free products and has also been investigated as a carrier for bioactive compounds [106]. These applications provide a practical basis for evaluating the incorporation of the inulin-containing powders into selected food matrices. However, the present study did not assess the nutritional or physiological effects of inulin itself.

Heat-processed foods, particularly bakery products, represent a relevant matrix for further evaluation. The carrier components themselves should not necessarily be regarded as chemically inert during thermal processing [75,107]. PPI provides reactive amino groups, whereas reducing termini of carbohydrate carriers such as maltodextrin and inulin can participate directly in Maillard-driven protein glycation; covalent PPI–maltodextrin [107] and PPI–inulin [75] conjugates have been demonstrated under controlled thermal conditions.

Evidence from bakery models indicates that plant-derived extracts and individual phenolic compounds can influence AGE formation during baking. Fortification of cookie dough with rooibos extract (0.5–1.0%, *w/w*) reduced several reactive dicarbonyls, including methylglyoxal and glyoxal, as well as specific non-fluorescent AGEs after baking [108]. In a separate cookie model, rosmarinic acid, an important phenolic constituent of *M. piperita*, showed substantial stability during baking, with 73.4% remaining after 10 min at 190 °C and reduced fluorescent AGE accumulation in the baked cookies [109]. This observation is relevant to peppermint because rosmarinic acid contributes to its in vitro antiglycation activity, although direct MGO trapping by this compound was not observed. In contrast, eriocitrin and eriodictyol formed MGO adducts in cell-free assays [9]. However, the magnitude of AGE inhibition reported across bakery models varies widely, ranging from less than 10% to more than 90% depending on the formulation, processing conditions, experimental system, and analytical endpoint [110]. Therefore, the effects observed in the present cell-free models cannot be assumed to persist after incorporation of the powders into thermally processed foods. Relevant AGE markers would need to be measured directly in the final product.

These application considerations and methodological limitations identify several priorities for further research. Although the low water activity of most carrier-containing powders may favor their preservation, storage stability was not evaluated. Packaging-dependent changes in moisture content, water activity, color, TPC, and antioxidant activity have been reported during six-month storage of another spray-dried encapsulated plant extract [111]. The present formulations should therefore be evaluated under defined storage and packaging conditions by monitoring moisture-related properties, color, phenolic composition, antioxidant capacity, and apparent antiglycation activity. Chromatographic identification and quantification of individual phenolic compounds would further clarify whether the differences among botanical materials, drying methods, and carrier formulations reflect changes in specific constituents.

Because antiglycation activity was assessed using cell-free fluorescence-based models, future studies should include sample-specific interference controls and complementary non-fluorescence-based analyses. Determination of residual methylglyoxal by HPLC and identification of phenolic–carbonyl adducts by mass spectrometry, as applied by Wang et al. (2011), could provide evidence of dicarbonyl trapping and help clarify the mechanisms underlying the observed apparent inhibition [68]. Simulated gastrointestinal digestion could subsequently be used to examine the release and bioaccessibility of phenolic compounds, as reviewed for spray-dried fruit-derived bioactives by Jafari et al. (2023) [25]. Appropriately selected cell-based models could then provide preliminary information on cellular uptake and biological effects. Finally, the formulations should be evaluated individually in relevant food matrices, with attention to matrix interactions, dispersibility, particle-related mouthfeel, herbal aroma, taste, and overall sensory acceptability.

Key findings:

• All powders showed apparent inhibition of fluorescent AGE formation in all four models. Carrier-free powders exceeded carrier-containing formulations by 16.9–37.9 percentage points, partly because equal masses of final powder contained different amounts of herbal solids.
• The responses were model- and concentration-dependent: MP was the most active botanical material in the BSA–Glu model, whereas AV was the most active in the BSA–MGO model. Neither drying method was consistently superior across all four models.
• AV showed measurable apparent inhibition in all four models; however, the results are limited to fluorescent endpoints in accelerated cell-free systems and cannot be extrapolated directly to antiglycation efficacy during food processing or in vivo.

## 4. Conclusions

This study identified trade-offs between drying techniques and carrier formulation rather than a single optimal powder. SD produced powders with lower moisture contents, whereas FD generally resulted in higher process efficiency; carrier-containing formulations generally had lower water activity and were easier to handle. Compared with carrier-containing formulations, carrier-free powders generally had higher TPC and antioxidant capacity per 100 g DM. Apparent inhibition of fluorescent AGE formation was likewise higher for carrier-free powders at the tested powder-equivalent concentrations. However, these comparisons were influenced by dilution of the herbal solids, and the effects of the carriers could not be assessed independently. *A. vulgaris* powders exhibited apparent inhibition of fluorescent AGE formation in all four glycation models. However, the botanical ranking was model-dependent, and neither drying technique was consistently superior. The assays assessed only fluorescent endpoints and did not include carrier-only controls or measurements of the undried formulations. As a result, retention of the observed response during powder production could not be determined. The impact of these powders on AGE formation during food processing remains unknown. The present data do not demonstrate in vivo antiglycation efficacy. Future studies in selected food matrices should directly assess storage stability, sensory acceptability, and relevant AGE markers.

## Figures and Tables

**Figure 1:**
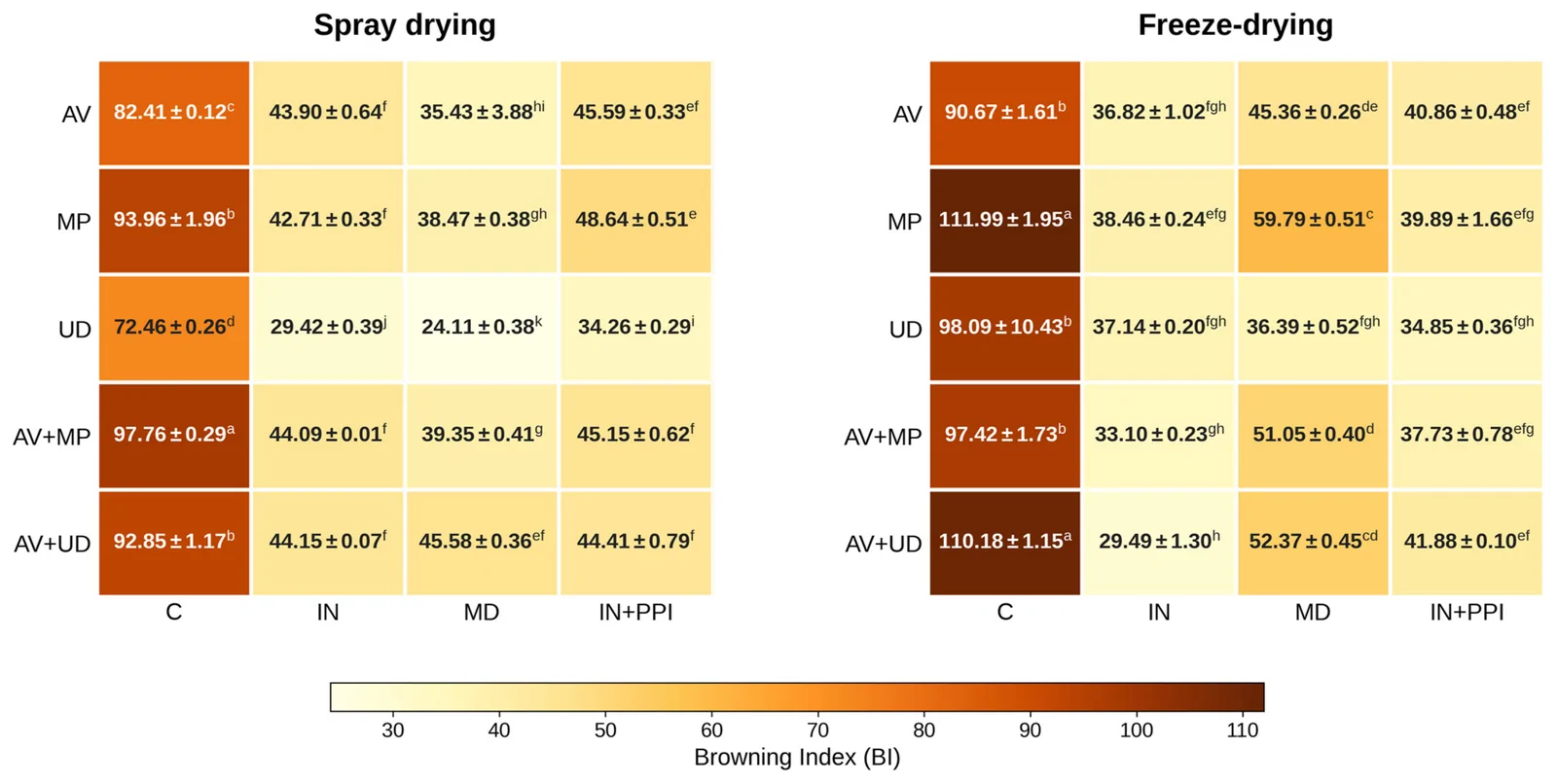
Browning index (BI) of spray-dried and freeze-dried herbal powders. Cell colors represent mean BI values; annotations show mean ± standard deviation calculated from three analytical color measurements (*n* = 3), not independent drying runs. Different superscript letters indicate significant differences among formulations and drying methods according to Tukey’s honestly significant difference (HSD) test (*p* < 0.05); values sharing at least one letter are not significantly different. AV—*Alchemilla vulgaris* L.; MP—*Mentha × piperita* L.; UD—*Urtica dioica* L.; AV + MP and AV + UD—binary herbal mixtures prepared at 1:1 (*w*/*w*); C—carrier-free control formulation; IN—inulin; MD—maltodextrin; IN + PPI—95:5 (*w*/*w*) inulin-pea-protein-isolate blend. BI was calculated from CIE L*a*b* coordinates and describes powder color rather than directly measuring chemical browning.

**Figure 2:**
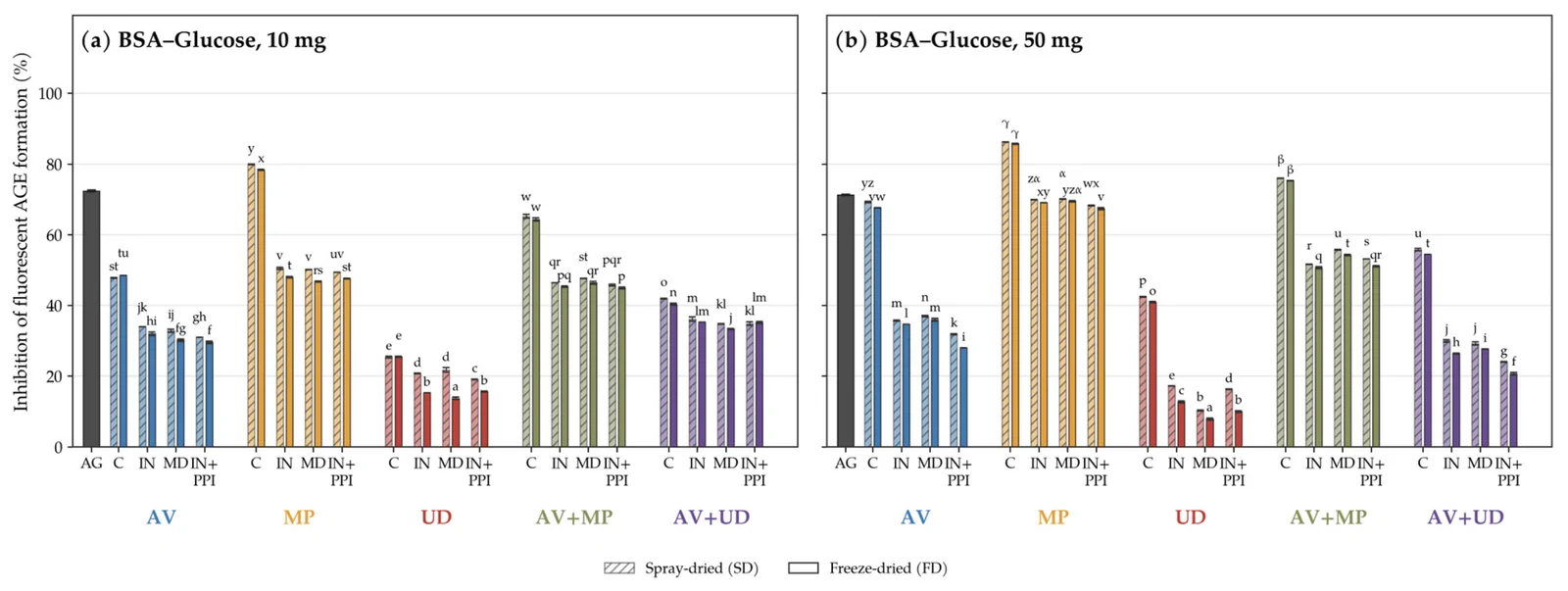
Inhibition of fluorescent AGE formation [%] by herbal powder extracts in the BSA–glucose model. Extracts were prepared from (**a**) 10 mg and (**b**) 50 mg of powder, corresponding to final reaction-mixture concentrations of 0.78 and 3.92 mg powder equivalents per mL, respectively. AG—aminoguanidine positive control; AV—*A. vulgaris*; MP—*M. piperita*; UD—*U. dioica*; C—carrier-free control formulation; IN—inulin; MD—maltodextrin; IN + PPI—inulin-pea-protein-isolate blend. Values are presented as mean ± SD from two independently prepared extracts, each measured in duplicate. Different letters indicate significant differences according to Tukey’s HSD test (*p* < 0.05). Greek letters (α, β, γ) continue the same lettering sequence once the Latin alphabet is used up.

**Figure 3:**
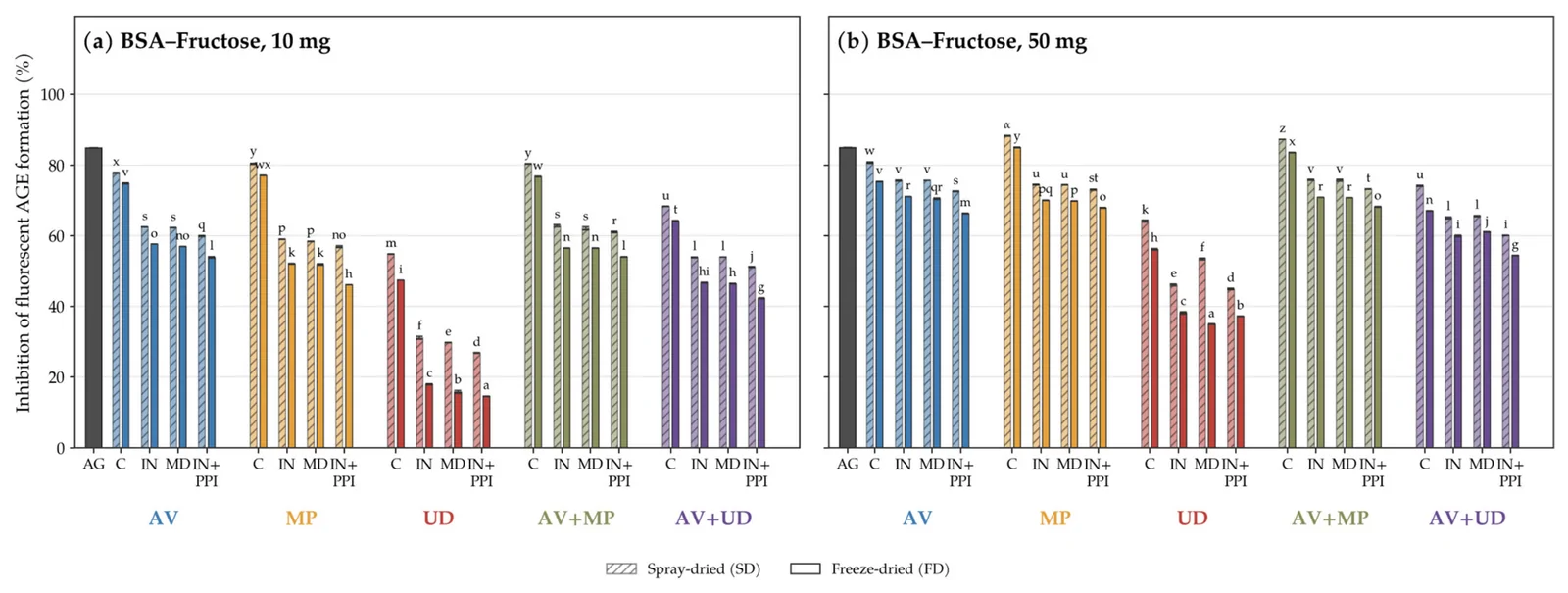
Inhibition of fluorescent AGE formation [%] by herbal powder extracts in the BSA–fructose model. Extracts were prepared from (**a**) 10 mg and (**b**) 50 mg of powder, corresponding to final reaction-mixture concentrations of 0.78 and 3.92 mg powder equivalents per mL, respectively. AG—aminoguanidine positive control; AV—*A. vulgaris*; MP—*M. piperita*; UD—*U. dioica*; C—carrier-free control formulation; IN—inulin; MD—maltodextrin; IN + PPI—inulin-pea-protein-isolate blend. Values are presented as mean ± SD from two independently prepared extracts, each measured in duplicate. Different letters indicate significant differences according to Tukey’s HSD test (*p* < 0.05). Greek letter (α) continues the same lettering sequence once the Latin alphabet is used up.

**Figure 4:**
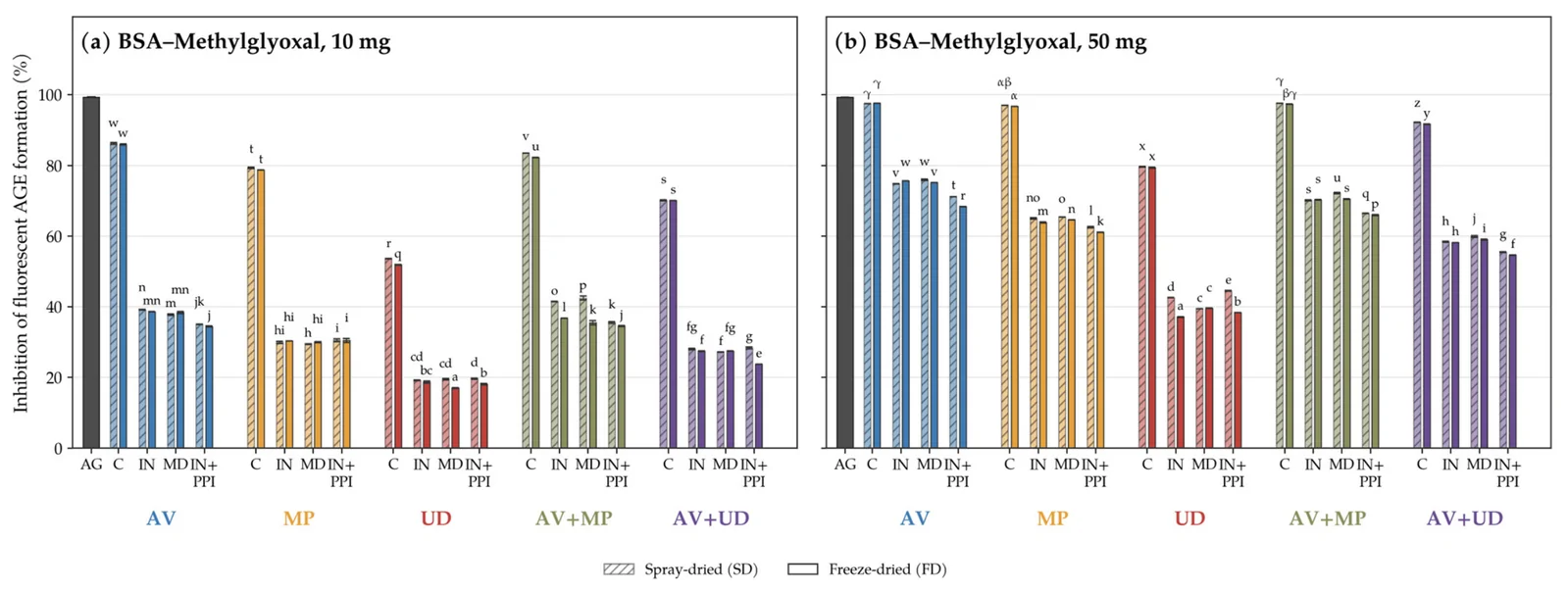
Inhibition of fluorescent AGE formation [%] by herbal powder extracts in the BSA–MGO model. Extracts were prepared from (**a**) 10 mg and (**b**) 50 mg of powder, corresponding to final reaction-mixture concentrations of 0.78 and 3.92 mg powder equivalents per mL, respectively. AG—aminoguanidine positive control; AV—*A. vulgaris*; MP—*M. piperita*; UD—*U. dioica*; C—carrier-free control formulation; IN—inulin; MD—maltodextrin; IN + PPI—inulin-pea-protein-isolate blend. Values are presented as mean ± SD from two independently prepared extracts, each measured in duplicate. Different letters indicate significant differences according to Tukey’s HSD test (*p* < 0.05). Greek letters (α, β, γ) continue the same lettering sequence once the Latin alphabet is used up.

**Figure 5:**
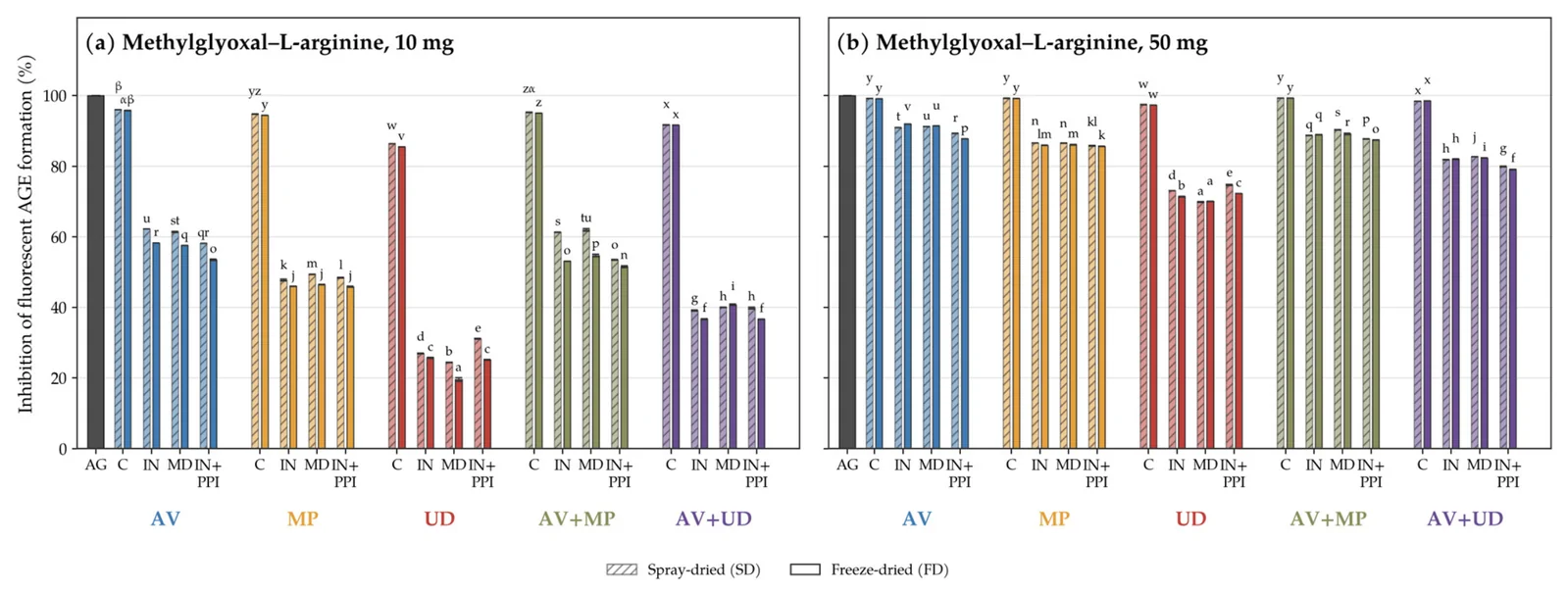
Inhibition of fluorescent AGE formation [%] by herbal powder extracts in the methylglyoxal–L-arginine model. Extracts were prepared from (**a**) 10 mg and (**b**) 50 mg of powder, corresponding to final reaction-mixture concentrations of 0.78 and 3.92 mg powder equivalents per mL, respectively. AG—aminoguanidine positive control; AV—*A. vulgaris*; MP—*M. piperita*; UD—*U. dioica*; C—carrier-free control formulation; IN—inulin; MD—maltodextrin; IN + PPI—inulin-pea-protein-isolate blend. Values are presented as mean ± SD from two independently prepared extracts, each measured in duplicate. Different letters indicate significant differences according to Tukey’s HSD test (*p* < 0.05). Greek letters (α, β) continue the same lettering sequence once the Latin alphabet is used up.

**Table 1:** Feed characteristics, spray drying outlet temperature, and process efficiency for both drying techniques.

Herb	Carrier	Brix	Spray Drying		Freeze-Drying
			Outlet Temperature	Process Efficiency	Process Efficiency
		(°Bx)	(°C)	(%)	(%)
AV	C	0.55 ± 0.06 ^e^	95–96	47.2 ± 2.2	91.1 ± 2.5
	IN	5.65 ± 0.07 ^a^	94	67.9 ± 1.7	87.0 ± 1.3
	MD	5.60 ± 0.00 ^ab^	98	76.9 ± 0.6	86.2 ± 1.6
	IN + PPI	4.60 ± 0.00 ^d^	94–95	76.8 ± 1.7	94.3 ± 1.3
MP	C	0.65 ± 0.06 ^e^	95–97	60.4 ± 2.4	89.3 ± 2.0
	IN	5.35 ± 0.07 ^c^	95	81.4 ± 3.7	87.2 ± 2.1
	MD	5.45 ± 0.07 ^abc^	97	79.0 ± 0.6	90.6 ± 0.4
	IN + PPI	4.60 ± 0.00 ^d^	95–96	76.1 ± 0.2	96.0 ± 0.2
UD	C	0.60 ± 0.00 ^e^	94–96	71.7 ± 3.5	84.9 ± 2.8
	IN	5.30 ± 0.00 ^c^	95	78.9 ±1.5	88.9 ± 1.0
	MD	5.40 ± 0.00 ^bc^	96–97	73.1 ± 0.2	96.8 ± 0.5
	IN + PPI	4.55 ± 0.07 ^d^	94–95	72.9 ± 1.3	92.0 ± 1.0
AV + MP	C	0.60 ± 0.12 ^e^	94–96	59.1 ± 1.3	98.1 ± 1.2
	IN	5.50 ± 0.00 ^abc^	95–96	82.0 ± 0.3	93.9 ± 0.4
	MD	5.40 ± 0.00 ^bc^	97	72.5 ± 0.4	98.0 ± 0.3
	IN + PPI	4.50 ± 0.00 ^d^	94	78.7 ± 1.0	94.8 ± 0.6
AV + UD	C	0.57 ± 0.05 ^e^	95–97	81.9 ± 3.5	74.8 ± 3.8
	IN	5.40 ± 0.00 ^bc^	94	81.4 ± 0.4	92.5 ± 1.1
	MD	5.30 ± 0.00 ^c^	97–98	81.4 ± 2.0	97.2 ± 1.5
	IN + PPI	4.40 ± 0.00 ^d^	96–97	81.2 ± 1.1	96.3 ± 1.1

Values are presented as mean ± standard deviation where applicable. Process efficiency was determined from two technological replicates for spray drying (*n* = 2) and three technological replicates for freeze-drying (*n* = 3). Spray drying outlet temperatures are reported as the observed values or ranges during processing. Different superscript letters within the °Brix column denote significant differences according to Tukey’s honestly significant difference (HSD) test (*p* < 0.05); values sharing at least one letter are not significantly different. AV—*Alchemilla vulgaris* L., MP—*Mentha × piperita* L., UD—*Urtica dioica* L., C—carrier-free control formulation, IN—inulin, MD—maltodextrin, IN + PPI—inulin-pea-protein-isolate blend.

**Table 2:** Moisture content and water activity of spray-dried or freeze-dried herbal powders.

Herb	Carrier	Spray Drying		Freeze-Drying	
		Moisture Content	Water Activity	Moisture Content	Water Activity
		(%)	(-)	(%)	(-)
AV	C	1.27 ± 0.16 ^a^	0.366 ± 0.000 ^no^	9.01 ± 0.27 ^rs^	0.339 ± 0.003 ^mn^
	IN	1.80 ± 0.25 ^abc^	0.139 ± 0.009 ^abcd^	4.32 ± 0.08 ^ijk^	0.165 ± 0.002 ^cdefg^
	MD	3.18 ± 0.06 ^fgh^	0.150 ± 0.001 ^bcde^	4.91 ± 0.06 ^kl^	0.170 ± 0.008 ^efg^
	IN + PPI	2.76 ± 0.12 ^defg^	0.179 ± 0.002 ^fgh^	5.66 ± 0.08 ^mno^	0.187 ± 0.006 ^gh^
MP	C	1.32 ± 0.12 ^a^	0.270 ± 0.014 ^kl^	10.44 ± 0.38 ^uv^	0.412 ± 0.005 ^q^
	IN	3.39 ± 0.14 ^gh^	0.225 ± 0.008 ^ij^	7.28 ± 0.19 ^q^	0.290 ± 0.002 ^l^
	MD	3.43 ± 0.47 ^gh^	0.131 ± 0.006 ^ab^	6.14 ± 0.11 ^o^	0.235 ± 0.007 ^ij^
	IN + PPI	2.67 ± 0.07 ^def^	0.169 ± 0.011 ^efg^	5.29 ± 0.09 ^lmn^	0.173 ± 0.009 ^efg^
UD	C	2.78 ± 0.00 ^defg^	0.225 ± 0.000 ^ij^	9.49 ± 0.14 st	0.497 ± 0.009 ^r^
	IN	4.18 ± 0.18 ^ij^	0.242 ± 0.001 ^jk^	6.21 ± 0.27 ^o^	0.224 ± 0.003 ^ij^
	MD	3.10 ± 0.07 ^efgh^	0.167 ± 0.015 ^defg^	5.98 ± 0.37 ^no^	0.248 ± 0.004 ^jk^
	IN + PPI	1.81 ± 0.03 ^abc^	0.116 ± 0.006 ^a^	4.59 ± 0.12 ^jkl^	0.118 ± 0.005 ^a^
AV + MP	C	1.48 ± 0.07 ^ab^	0.284 ± 0.009 ^l^	9.74 ± 0.18 ^tu^	0.498 ± 0.002 ^r^
	IN	3.69 ± 0.01 ^hi^	0.241 ± 0.008 ^jk^	6.95 ± 0.06 ^pq^	0.232 ± 0.004 ^ij^
	MD	2.40 ± 0.03 ^cde^	0.134 ± 0.009 ^ab^	8.31 ± 0.11 ^r^	0.382 ± 0.010 ^op^
	IN + PPI	2.06 ± 0.22 ^bcd^	0.120 ± 0.008 ^a^	5.26 ± 0.01 ^lm^	0.180 ± 0.003 ^fgh^
AV + UD	C	1.13 ± 0.08 ^a^	0.239 ± 0.002 ^j^	10.49 ± 0.14 ^v^	0.403 ± 0.006 ^pq^
	IN	3.16 ± 0.06 ^fgh^	0.207 ± 0.007 ^hi^	6.27 ± 0.17 ^op^	0.225 ± 0.003 ^ij^
	MD	2.63 ± 0.00 ^def^	0.156 ± 0.013 ^bcdef^	7.41 ± 0.16 ^q^	0.323 ± 0.003 ^m^
	IN + PPI	2.48 ± 0.00 ^cdef^	0.158 ± 0.008 ^bcdef^	4.88 ± 0.19 ^jkl^	0.136 ± 0.005 ^abc^

Values are presented as mean ± standard deviation (*n* = 2). Different superscript letters within the same parameter denote significant differences according to Tukey’s honestly significant difference (HSD) test (*p* < 0.05); values sharing at least one letter are not significantly different. AV—*Alchemilla vulgaris* L., MP—*Mentha × piperita* L., UD—*Urtica dioica* L., C—carrier-free control formulation, IN—inulin, MD—maltodextrin, IN + PPI—inulin-pea-protein-isolate blend.

**Table 3:** CIE L*a*b* color parameters of spray- and freeze-dried herbal powders.

Herb	Carrier	Spray Drying			Freeze-Drying		
		L*	a*	b*	L*	a*	b*
AV	C	50.11 ± 0.81 ^i^	3.99 ± 0.10 ^c^	27.86 ± 0.42 ^b^	52.51 ± 0.53 ^g^	5.63 ± 0.14 ^c^	30.79 ± 0.18 ^a^
	IN	61.20 ± 0.71 ^fg^	1.45 ± 0.18 ^fg^	21.69 ± 0.10 ^gh^	74.62 ± 0.40 ^bc^	1.09 ± 0.09 ^i^	23.11 ± 0.40 ^ef^
	MD	69.83 ± 1.03 ^bc^	0.66 ± 0.55 ^i^	21.09 ± 1.40 ^hi^	72.01 ± 0.69 ^d^	1.80 ± 0.06 ^h^	26.19 ± 0.33 ^c^
	IN + PPI	59.84 ± 0.81 ^g^	1.76 ± 0.09 ^f^	21.72 ± 0.27 ^fgh^	77.72 ± 0.90 ^a^	1.27 ± 0.05 ^i^	26.23 ± 0.08 ^c^
MP	C	44.22 ± 0.83 ^j^	6.63 ± 0.11 ^a^	25.92 ± 0.10 ^c^	37.65 ± 0.66 ^h^	9.07 ± 0.02 ^a^	24.06 ± 0.14 ^def^
	IN	69.05 ± 0.63 ^bcd^	3.37 ± 0.06 ^d^	22.99 ± 0.21 ^def^	72.40 ± 0.84 ^d^	3.44 ± 0.03 ^f^	21.96 ± 0.19 ^g^
	MD	67.90 ± 0.50 ^cde^	2.81 ± 0.06 ^e^	20.72 ± 0.21 ^hij^	63.14 ± 0.89 ^f^	5.85 ± 0.01 ^c^	26.71 ± 0.22 ^c^
	IN + PPI	54.76 ± 1.01 ^h^	3.58 ± 0.05 ^cd^	19.89 ± 0.28 ^ij^	71.37 ± 0.41 ^d^	3.60 ± 0.23 ^f^	22.27 ± 0.60 ^g^
UD	C	47.55 ± 0.62 ^i^	0.11 ± 0.01 ^j^	25.50 ± 0.27 ^c^	36.75 ± 0.36 ^hi^	0.41 ± 0.03 ^j^	24.07 ± 1.52 ^def^
	IN	73.43 ± 0.69 ^a^	−1.61 ± 0.05 ^k^	20.26 ± 0.07 ^ij^	74.08 ± 0.69 ^bc^	−3.25 ± 0.04 ^n^	25.53 ± 0.14 ^c^
	MD	69.91 ± 1.70 ^bc^	−2.11 ± 0.07 ^l^	16.75 ± 0.20 ^k^	74.78 ± 0.16 ^bc^	−2.21 ± 0.01 ^m^	24.78 ± 0.29 ^d^
	IN + PPI	62.72 ± 0.93 ^f^	−1.44 ± 0.03 ^k^	19.59 ± 0.22 ^j^	73.60 ± 0.35 ^c^	−3.53 ± 0.02 ^o^	24.34 ± 0.08 ^de^
AV + MP	C	45.59 ± 0.94 ^j^	6.42 ± 0.15 ^a^	27.72 ± 0.53 ^b^	35.61 ± 0.84 ^i^	8.20 ± 0.13 ^b^	20.44 ± 0.30 ^h^
	IN	66.10 ± 0.15 ^e^	2.81 ± 0.01 ^e^	22.86 ± 0.05 ^efg^	75.76 ± 0.70 ^b^	1.73 ± 0.01 ^h^	21.04 ± 0.08 ^gh^
	MD	70.92 ± 1.31 ^ab^	2.49 ± 0.03 ^e^	22.45 ± 0.29 ^efg^	67.75 ± 1.00 ^e^	4.21 ± 0.12 ^e^	25.90 ± 0.44 ^c^
	IN + PPI	59.23 ± 0.29 ^g^	2.56 ± 0.09 ^e^	20.68 ± 0.26 ^hij^	74.86 ± 0.94 ^bc^	2.53 ± 0.11 ^g^	22.88 ± 0.09 ^f^
AV + UD	C	49.07 ± 1.32 ^i^	4.84 ± 0.11 ^b^	29.45 ± 0.60 ^a^	32.08 ± 0.17 ^j^	4.93 ± 0.05 ^d^	21.12 ± 0.05 ^gh^
	IN	66.55 ± 0.40 ^de^	0.65 ± 0.01 ^i^	24.19 ± 0.13 ^d^	79.04 ± 0.57 ^a^	−0.49 ± 0.11 ^l^	21.11 ± 0.66 ^gh^
	MD	62.72 ± 0.40 ^f^	1.14 ± 0.04 ^gh^	23.03 ± 0.23 ^de^	68.99 ± 0.16 ^e^	1.61 ± 0.08 ^h^	28.31 ± 0.12 ^b^
	IN + PPI	62.30 ± 0.50 ^f^	0.78 ± 0.07 ^hi^	22.67 ± 0.22 ^efg^	74.43 ± 0.34 ^bc^	0.12 ± 0.02 ^k^	26.24 ± 0.09 ^c^

Values are presented as mean ± standard deviation (*n* = 3). For each color parameter (L^*^, a^*^, b^*^), different superscript letters denote significant differences among formulations and drying methods according to one-way ANOVA followed by Tukey’s HSD test (*p* < 0.05). Values sharing at least one letter are not significantly different. AV—*Alchemilla vulgaris* L., MP—*Mentha × piperita* L., UD—*Urtica dioica* L., C—carrier-free control formulation, IN—inulin, MD—maltodextrin, IN + PPI—inulin-pea-protein-isolate blend.

**Table 4:** Total phenolics content and antioxidant capacity of spray- and freeze-dried herbal powders determined in methanolic extracts.

Herb	Carrier	Spray Drying				Freeze-Drying			
		TPC	Fast Blue	TEAC ABTS	FRAP	TPC	Fast Blue	TEAC ABTS	FRAP
		(g GAE/100 g DM)		(mmol TE/100 g DM)		(g GAE/100 g DM)		(mmol TE/100 g DM)	
AV	C	28.65 ± 0.79 ^r^	23.55 ± 0.18 ^u^	228.58 ± 3.38 ^u^	214.95 ± 0.54 ^y^	36.99 ± 0.47 ^s^	26.10 ± 0.05 ^v^	307.47 ± 1.49 ^v^	170.73 ± 0.80 ^x^
	IN	3.31 ± 0.02 ^hijk^	2.41 ± 0.02 ^f^	23.11 ± 0.42 ^k^	23.13 ± 0.22 ^q^	3.47 ± 0.05 ^ijkl^	3.07 ± 0.01 ^kl^	36.96 ± 0.10 ^n^	21.18 ± 0.01 ^p^
	MD	3.44 ± 0.05 ^ijkl^	3.18 ± 0.03 ^lm^	18.00 ± 0.02 ^ij^	17.55 ± 0.38 ^n^	3.46 ± 0.18 ^ijkl^	3.44 ± 0.05 ^mn^	32.87 ± 0.05 ^m^	18.74 ± 0.11 ^o^
	IN + PPI	3.62 ± 0.03 ^jkl^	2.51 ± 0.08 ^fg^	25.09 ± 0.16 ^kl^	22.62 ± 0.14 ^q^	3.66 ± 0.04 ^kl^	2.66 ± 0.04 ^fghi^	35.70 ± 0.28 ^n^	21.07 ± 0.01 ^p^
MP	C	15.41 ± 0.15 ^o^	20.97 ± 0.01 ^r^	55.36 ± 0.51 ^p^	69.38 ± 0.36 ^u^	15.04 ± 0.21 ^o^	21.33 ± 0.06 ^s^	123.21 ± 0.44 ^s^	66.06 ± 0.20 ^t^
	IN	1.99 ± 0.13 ^cdef^	2.61 ± 0.18 ^fghi^	7.98 ± 0.19 ^e^	8.46 ± 0.04 ^gh^	1.79 ± 0.00 ^cde^	2.53 ± 0.18 ^fgh^	13.93 ± 0.09 ^fgh^	7.17 ± 0.02 ^ef^
	MD	1.75 ± 0.01 ^cd^	2.87 ± 0.01 ^hijkl^	3.86 ± 0.09 ^bc^	5.52 ± 0.01 ^d^	1.66 ± 0.03 ^cd^	2.89 ± 0.00 ^ijkl^	14.06 ± 0.05 ^fgh^	7.10 ± 0.02 ^ef^
	IN + PPI	1.83 ± 0.01 ^cde^	2.50 ± 0.07 ^fg^	7.36 ± 0.05 ^de^	7.40 ± 0.05 ^ef^	1.78 ± 0.01 ^cde^	2.39 ± 0.07 ^f^	13.90 ± 0.03 ^fgh^	6.97 ± 0.09 ^e^
UD	C	4.12 ± 0.13 ^l^	4.64 ± 0.01 ^o^	13.78 ± 0.65 ^fgh^	16.10 ± 0.03 ^m^	3.31 ± 0.01 ^hijk^	3.64 ± 0.02 ^n^	20.22 ± 0.11 ^j^	7.83 ± 0.01 ^fg^
	IN	0.83 ± 0.01 ^ab^	1.37 ± 0.02 ^cd^	2.53 ± 0.00 ^ab^	3.53 ± 0.01 ^c^	0.77 ± 0.01 ^ab^	0.89 ± 0.00 ^ab^	5.32 ± 0.00 ^cd^	2.80 ± 0.01 ^bc^
	MD	0.77 ± 0.02 ^ab^	0.81 ± 0.00 ^ab^	0.94 ± 0.01 ^a^	1.70 ± 0.02 ^a^	0.81 ± 0.02 ^ab^	1.11 ± 0.03 ^bc^	3.40 ± 0.03 ^abc^	1.83 ± 0.00 ^a^
	IN + PPI	0.84 ± 0.02 ^ab^	0.72 ± 0.04 ^a^	2.72 ± 0.16 ^abc^	3.49 ± 0.00 ^c^	0.63 ± 0.02 ^a^	0.73 ± 0.01 ^a^	4.90 ± 0.02 ^bcd^	2.52 ± 0.02 ^ab^
AV + MP	C	22.98 ± 0.10 ^q^	19.39 ± 0.06 ^q^	110.11 ± 0.23 ^r^	100.97 ± 0.00 ^w^	21.12 ± 0.53 ^p^	22.25 ± 0.12 ^t^	179.96 ± 0.35 ^t^	98.55 ± 0.00 ^v^
	IN	2.74 ± 0.02 ^fghi^	2.81 ± 0.00 ^ghijk^	17.14 ± 0.26 ^i^	16.22 ± 0.01 ^m^	2.89 ± 0.02 ^ghij^	2.72 ± 0.00 ^fghij^	26.91 ± 0.05 ^l^	14.12 ± 0.01 ^l^
	MD	2.60± 0.01 ^fgh^	2.77 ± 0.01 ^ghijk^	7.45 ± 0.05 ^de^	9.61 ± 0.10 ^i^	2.63 ± 0.01 ^fgh^	3.04 ± 0.01 ^jkl^	24.78 ± 0.03 ^kl^	13.79 ± 0.01 ^kl^
	IN + PPI	2.38 ± 0.01 ^defg^	2.72 ± 0.33 ^fghij^	15.53 ± 0.11 ^hi^	16.35 ± 0.01 ^m^	2.52 ± 0.01 ^efg^	2.41 ± 0.12 ^f^	24.53 ± 0.15 ^kl^	13.21 ± 0.01 ^k^
AV + UD	C	12.16 ± 0.15 ^n^	11.66 ± 0.01 ^p^	52.07 ± 0.76 ^o^	50.97 ± 0.22 ^s^	11.11 ± 0.04 ^m^	11.73 ± 0.01 ^p^	71.54 ± 0.18 ^q^	44.43 ± 0.33 ^r^
	IN	1.83 ± 0.03 ^cde^	1.45 ± 0.01 ^cd^	12.21 ± 0.03 ^fg^	11.54 ± 0.04 ^j^	1.84 ± 0.03 ^cde^	1.47 ± 0.07 ^d^	15.82 ± 0.02 ^hi^	8.84 ± 0.02 ^hi^
	MD	1.46 ± 0.02 ^bc^	1.62 ± 0.02 ^de^	7.16 ± 0.03 ^de^	7.47 ± 0.03 ^ef^	1.75 ± 0.01 ^cd^	1.91 ± 0.01 ^e^	11.74 ± 0.03 ^fg^	7.01 ± 0.03 ^e^
	IN + PPI	1.81 ± 0.00 ^cde^	1.52 ± 0.00 ^d^	11.56 ± 0.03 ^f^	10.81 ± 0.05 ^j^	1.63 ± 0.07 ^cd^	1.47 ± 0.06 ^d^	14.29 ± 0.05 ^gh^	8.35 ± 0.06 ^gh^

DM—Dry Matter, TPC—Total Phenolics Content, TEAC ABTS—Trolox Equivalent Antioxidant Capacity by ABTS, FRAP—Ferric Reducing Antioxidant Power, GAE—Gallic Acid Equivalents, TE—Trolox Equivalents, AV—*Alchemilla vulgaris* L., MP—*Mentha × piperita* L., UD—*Urtica dioica* L., C—carrier-free control formulation, IN—inulin, MD—maltodextrin, IN + PPI—inulin with pea protein isolate. Values are presented as mean ± standard deviation of two independently prepared methanolic extracts (*n* = 2); technical duplicates were averaged within each extract prior to calculating the mean and standard deviation across extracts. Different superscript letters within the same column indicate significant differences according to Tukey’s HSD test (*p* < 0.05); values sharing at least one letter are not significantly different.

**Figure A1:**
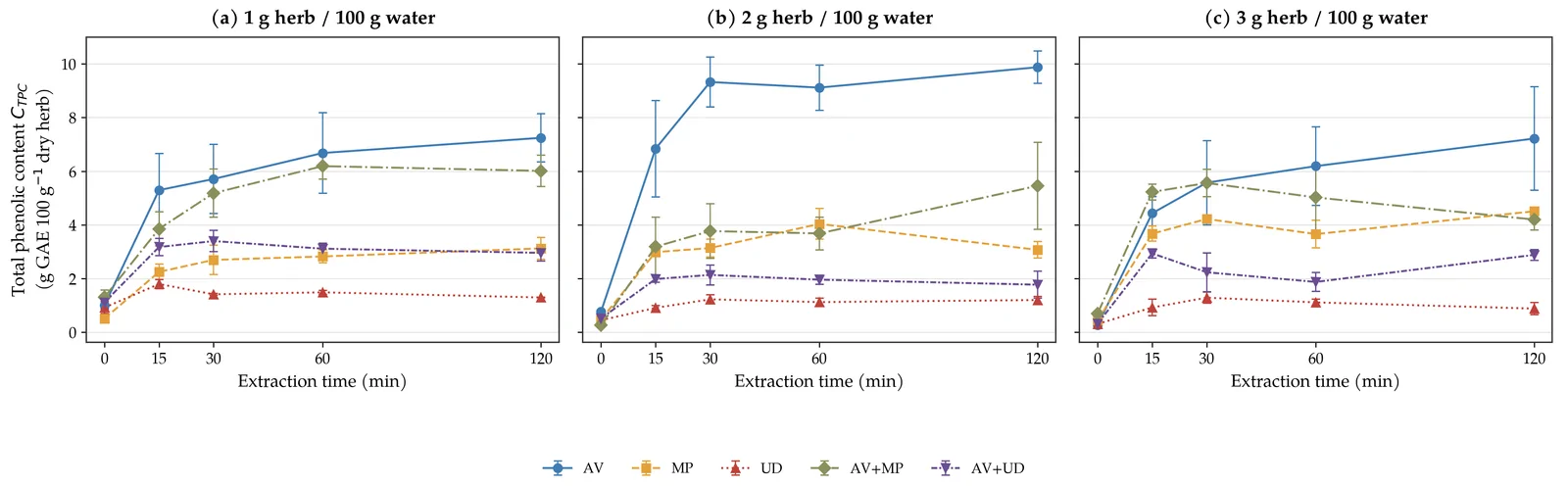
Effect of extraction time and herb/water ratio on the total phenolics content (TPC) of aqueous herbal extracts. Extractions were performed at 90 °C using (**a**) 1 g, (**b**) 2 g, or (**c**) 3 g of dried herbal material per 100 g of water. AV—*A. vulgaris*; MP—*M. piperita*; UD—*U. dioica*; AV + MP—*A. vulgaris* + *M. piperita* (1:1, *w/w*); AV + UD—*A. vulgaris* + *U. dioica* (1:1, *w/w*). Data are presented as mean ± standard deviation of two independent extractions (*n* = 2), each analyzed in duplicate.

## Data Availability

The data presented in this study are available on request from the corresponding author.

## Abbreviations

ABTS	2,2′-azino-bis(3-ethylbenzothiazoline-6-sulfonic acid) diammonium salt
AG	Aminoguanidine (positive control in the antiglycation assays)
AGE(s)	Advanced glycation end product(s)
ANOVA	Analysis of variance
AV	*Alchemilla vulgaris* L.
BI	Browning index
BSA	Bovine serum albumin
BSA–Fru	BSA–fructose (glycation model)
BSA–Glu	BSA–glucose (glycation model)
BSA–MGO	BSA–methylglyoxal (glycation model)
C	Carrier-free control formulation (with water instead of carrier)
DM	Dry matter
DPPH	2,2-diphenyl-1-picrylhydrazyl
FD	Freeze drying
FRAP	Ferric reducing antioxidant power
GAE	Gallic acid equivalent(s)
HSD	Honestly significant difference (Tukey’s HSD test)
IN	Inulin
MD	Maltodextrin
MGO	Methylglyoxal
MGO–Arg	Methylglyoxal–L-arginine (glycation model)
MP	*Mentha* × *piperita* L.
PPI	Pea protein isolate
SD	Spray drying
TE	Trolox equivalent(s)
TEAC	Trolox equivalent antioxidant capacity
TPTZ	2,4,6-Tris(2-pyridyl)-s-triazine
TPC	Total phenolics content
UD	*Urtica dioica* L.

## Appendix A

### Appendix A.1. Preliminary Selection of Aqueous Extraction Conditions

Preliminary extraction trials were conducted to compare the effects of extraction time and herb/water ratio on the total phenolics content (TPC) of the aqueous herbal extracts. Two independent extracts were prepared for each experimental condition, and each extract was analyzed in duplicate. Technical replicates were averaged within each independent extract, after which the mean and standard deviation were calculated from the two independent extract means. Owing to the limited number of independent extractions, the results were evaluated descriptively and no inferential statistical analysis was performed.

### Appendix A.2. Gravimetric Determination of Feed Total Solids

The total solids content of each feed formulation (carrier-free extract or extract combined with a carrier) was determined gravimetrically before drying. Total solids content was calculated from the mass of the dry residue relative to the initial feed mass and expressed as g/100 g feed. Each formulation was analyzed in duplicate (*n* = 2). Because only duplicate determinations were available, the results were summarized descriptively, and no inferential statistical analysis was performed. These values were used to calculate process efficiency (Section 2.10) and, together with the known amount of added carrier, to estimate the carrier-derived fraction of the initial feed solids.

**Table A1:** Gravimetrically determined total solids content of the feed formulations prior to drying.

Botanical System	C	IN	MD	IN + PPI
AV	0.75 ± 0.04	5.47 ± 0.13	5.35 ± 0.04	5.57 ± 0.12
MP	0.65 ± 0.03	5.52 ± 0.25	5.25 ± 0.04	5.51 ± 0.02
UD	0.61 ± 0.03	5.50 ± 0.10	5.18 ± 0.01	5.64 ± 0.10
AV + MP	0.65 ± 0.01	5.48 ± 0.02	5.29 ± 0.03	5.49 ± 0.07
AV + UD	0.46 ± 0.02	5.44 ± 0.03	5.18 ± 0.13	5.33 ± 0.07

Values are presented as mean ± standard deviation of duplicate determinations (*n* = 2) and expressed as g/100 g feed. AV—*Alchemilla vulgaris* L.; MP—*Mentha* × *piperita* L.; UD—*Urtica dioica* L.; C—carrier-free control formulation; IN—inulin; MD—maltodextrin; IN + PPI—95:5 (*w/w*) inulin–pea protein isolate blend. AV + MP and AV + UD were 1:1 (*w/w*) herbal mixtures.
